# CSAG-DETR: a lightweight detector for accurate weed detection in sugar beet fields under complex field conditions

**DOI:** 10.3389/fpls.2026.1875124

**Published:** 2026-08-03

**Authors:** Xucong Luo, Yisa Watbek, Junyi Lv, Chen Li, Reyim Mamut, Guldiyar Adil, Yurong Qian

**Affiliations:** 1 Xinjiang Engineering Research Center of Big Data and Intelligent Software, School of Software, Xinjiang University, Urumqi, China; 2 Key Laboratory of Software Engineering, Xinjiang University, Urumqi, China; 3 School of Computer Science and Technology, Xinjiang University, Urumqi, China; 4 School of Information Engineering, Changji University, Changji, China; 5 College of Life Sciences and Technology, Xinjiang University, Urumqi, China; 6 International Joint Laboratory of Multilingual Cognitive Computing Along the Silk Road, Urumqi, China

**Keywords:** CSAG-DETR, deep learning, object detection, precision agriculture, sugar beet weed detection

## Abstract

**Introduction:**

Effective weed management is essential for reducing yield losses in sugar beet cultivation. However, existing deep learning-based detectors often experience performance degradation in complex field environments characterized by variable illumination, severe occlusion, dense vegetation, and background interference. In addition, models lacking lightweight designs generally require substantial computational resources, leading to increased inference latency and deployment costs that limit their application in real-time agricultural systems.

**Methods:**

To address these challenges, we developed CSAG-DETR, a lightweight and robust weed detection framework based on RT-DETR-R18 for accurate weed detection in sugar beet fields. The proposed framework incorporates a Cross-Stage Multi-Scale Network (CSMN) to enhance hierarchical feature interaction, a Cross-Stage Local-Detail Module (CSLM) to preserve fine-grained textures and object boundaries, and Global Attention-Gated Dual-Path Upsampling and Downsampling modules (GAGDU and GAGDD) to improve the stability of cross-scale feature transformation. Furthermore, we constructed the BeetWeed dataset containing nine common weed species and applied diverse data augmentation strategies to improve model robustness under variable field conditions.

**Results:**

Experimental results showed that CSAG-DETR achieved an mAP@0.5 of 98.9%, an mAP@0.5:0.95 of 78.6%, and an inference speed of 167.3 FPS on the BeetWeed dataset, outperforming 13 mainstream object detection models in terms of overall detection performance and computational efficiency. Generalization experiments conducted on the public CottonWeedDet12 dataset further demonstrated the strong cross-dataset generalization capability and competitive overall performance of the proposed model.

**Discussion:**

These results indicate that CSAG-DETR effectively balances detection accuracy, inference efficiency, and robustness in complex agricultural environments. The proposed framework therefore provides a practical solution for real-time weed detection and may support the deployment of intelligent weed management systems in sugar beet production.

## 1 Introduction

The persistent challenge of weed infestation in modern agricultural systems poses a serious threat to global food security. Weeds are estimated to account for nearly 43% of annual crop yield losses worldwide (Li et al., 2024a), causing substantial economic damage and impeding efforts to meet the growing food demands of an expanding population (Sassano et al., 2025). Among the affected crops, sugar beet, an important raw material for sugar production and bioenergy, is particularly vulnerable to weed competition throughout its growth cycle (Tang et al., 2025). By competing aggressively for water, nutrients, and sunlight, weeds suppress crop growth and may also serve as reservoirs for pests and pathogens, thereby reducing yield and root quality and threatening the sustainability of the sugar industry (Li and Zhang, 2024).

Conventional approaches to weed management, which primarily rely on manual scouting and visual checks, are excessively labor-demanding and costly, rendering them inadequate for contemporary precision agriculture and large-scale farming (Weihs et al., 2024). Driven by escalating challenges such as climate shifts, workforce deficits, and rigorous pesticide regulations, the creation of smart and precise weed identification systems has become crucial for sustaining sugar beet cultivation and securing global food supplies (Liu et al., 2024a). Nevertheless, deploying computer vision models in actual field settings faces multiple practical hurdles. Fluctuations in lighting, camera perspectives, and shadows can significantly disrupt image consistency and color stability (He et al., 2025). Furthermore, heavy field vegetation causes substantial leaf overlapping and occlusion, obstructing weeds from full view. Feature extraction is additionally compromised by motion blur, environmental noise, and partial blockages. Current detection frameworks also struggle to process the intricate spatial and semantic contexts inherent to chaotic field environments (Yan et al., 2023). Ultimately, these factors lead to inconsistent feature extraction and diminished accuracy, emphasizing the urgent demand for highly robust, context-sensitive visual algorithms designed specifically for complex agricultural conditions (Mao et al., 2025).

Beyond environmental challenges, agricultural deployment imposes rigorous demands on model efficiency. For systems utilizing edge computing, mobile platforms, and smart weeders, detection algorithms must deliver high accuracy while remaining compact enough to ensure real-time operation under limited hardware resources. Models burdened by excessive parameters and high computational complexity typically suffer from significant memory overhead, slow inference speeds, and elevated deployment costs, severely limiting their utility in precision agriculture. Consequently, designing a detector that strikes an optimal balance between robustness, accuracy, and computational efficiency is crucial for effective field-level weed management.

To boost the performance of precision agriculture, numerous studies have recently focused on computer vision applications for weed identification in diverse crops (Liu et al., 2025a). For example, deep learning algorithms, including CNNs and semantic segmentation, have been used to distinguish weeds from crop plants under variable field conditions. In addition, one-stage object detectors, especially the YOLO series, have been widely adopted in cotton, rice, and other crop scenarios because of their high inference speed, compact deployment pipeline, and strong suitability for real-time autonomous weeding systems (Li et al., 2025). These advantages make YOLO-type detectors attractive for edge devices and mobile agricultural platforms. However, YOLO-based methods still have several limitations in complex weed detection scenarios. Their detection performance is often affected by anchor or grid assignment strategies, nonmaximum suppression, and local convolution-dominated feature extraction, which may reduce robustness when weeds are small, densely distributed, heavily occluded, or visually similar to crop leaves. In sugar beet fields, where dense vegetation, irregular illumination, heterogeneous soil backgrounds, and severe crop–weed overlap frequently occur, these limitations may lead to missed detections or inaccurate localization of small and partially occluded weed targets. Compared with the YOLO series, DETR-based detectors reformulate object detection as an end-to-end set prediction problem and can capture long-range dependencies through transformer-based attention mechanisms. The RT-DETR series further improves the practical applicability of DETR-style models by reducing inference latency and enabling real-time detection while maintaining the advantages of global contextual modeling. Therefore, RT-DETR is potentially more suitable for complex agricultural scenes where weed recognition requires both local detail perception and global crop–weed contextual reasoning. Nevertheless, the standard RT-DETR series also has limitations. Its lightweight variants may lose fine-grained edge and texture information during repeated downsampling, whereas heavier variants can improve representation capacity but introduce higher computational cost, larger model size, and greater deployment burden.

Compared with the YOLO series, DETR-based detectors reformulate object detection as an end-to-end set prediction problem and can capture long-range dependencies through transformer-based attention mechanisms. The RT-DETR series further improves the practical applicability of DETR-style models by reducing inference latency and enabling real-time detection while maintaining the advantages of global contextual modeling. Therefore, RT-DETR is potentially more suitable for complex agricultural scenes where weed recognition requires both local detail perception and global crop–weed contextual reasoning. Nevertheless, the standard RT-DETR series also has limitations. Its lightweight variants may lose fine-grained edge and texture information during repeated downsampling, whereas heavier variants can improve representation capacity but introduce higher computational cost, larger model size, and greater deployment burden. Moreover, the original RT-DETR architecture is not specifically optimized for dense sugar beet fields, where small weeds, overlapping leaves, illumination variation, and background clutter require stronger multi-scale representation, local-detail preservation, and stable cross-scale feature transformation.

**Figure 1:**
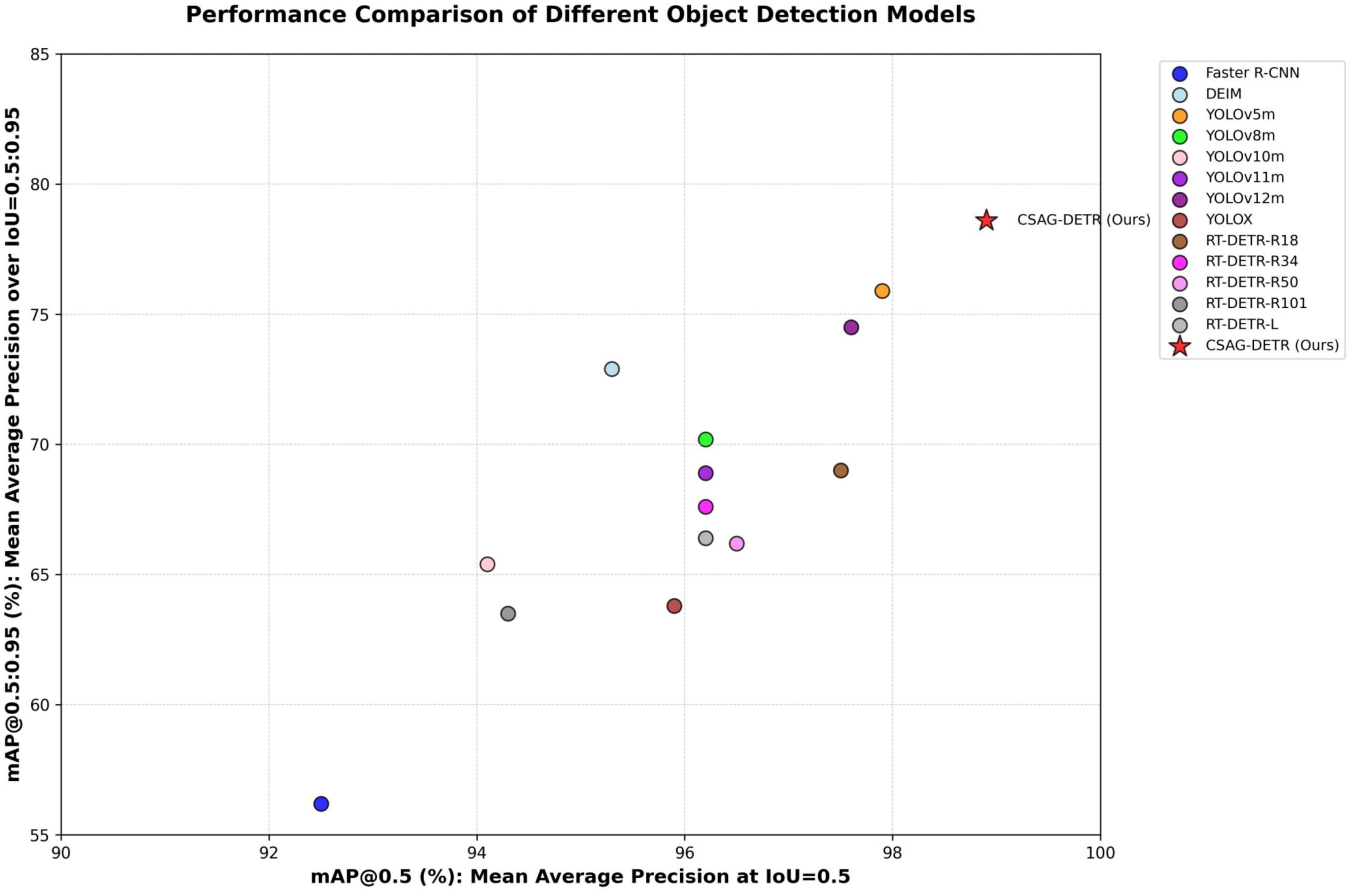
Comparison of different object detection algorithms on the BeetWeed dataset in terms of weed detection performance.

To overcome the aforementioned challenges, this paper introduces CSAG-DETR (Cross-Stage Attention Gated DETR), a streamlined, real-time object detection model designed specifically for identifying weeds in sugar beet crops. Utilizing the RT-DETR-R18 baseline, our proposed framework demonstrates exceptional detection capabilities on the BeetWeed dataset, even in challenging agricultural environments (see **Figure 1**). Ultimately, this research delivers an automated, high-efficiency approach to precision weed control, facilitating rapid weed identification, minimizing crop yield reduction, and promoting sustainable farming practices.

The main contributions of this study are summarized as follows:

- Under the annotation and guidance of agricultural specialists, we construct the BeetWeed dataset, which contains nine categories of sugar beet weed species collected from real field environments. The dataset provides a reliable benchmark for training and evaluating weed detection models under practical agricultural conditions.
- We design the Cross-Stage Multi-Scale Network (CSMN) to enhance hierarchical contextual representation in the backbone by integrating multi-scale adaptive pooling and edge-aware enhancement across cross-stage feature connections, thereby improving the detection of small-scale and occluded weeds.
- We introduce the Cross-Stage Local-Detail Module (CSLM), which combines small-dilation asymmetric atrous convolutions with a task-specific dual-branch structure to enhance high-frequency textures and boundary information while maintaining semantic stability.
- We further develop the Global Attention-Gated Dual-Path Upsampler (GAGDU) and Downsampler (GAGDD), two lightweight modules that improve semantic consistency and spatial structure preservation during multi-scale feature transitions through global-attention-guided channel gating and detail-aware dual-path refinement.

## 2 Related work

### 2.1 Traditional machine learning approaches for crop weed identification

Conventional machine learning approaches demonstrate significant explainability and robustness in the context of weed recognition among crops (Sun et al., 2024; Dong et al., 2025). These approaches generally follow a multi-stage pipeline, including vegetation segmentation or region proposal, handcrafted feature extraction, and classifier design. Commonly used discriminative descriptors include color indices, RGB and HSV features, vegetation indices, texture descriptors such as GLCM, LBP, and HOG, as well as shape moments and contour-based characteristics for distinguishing crops from weeds. Driven by advancements in spectral imaging and classical algorithms, a growing body of literature integrates hyperspectral data with conventional machine learning architectures (Liu et al., 2024b). By fusing morphological, textural, and spectral characteristics with standard classifiers—including support vector machines (SVM), random forests (RF), k-nearest neighbors (KNN), partial least squares discriminant analysis (PLS-DA), and shallow multilayer perceptrons (MLPs)—these synergistic methods yield dependable and transparent weed identification even in demanding agricultural environments (Wang et al., 2024).

As an illustration, Zhang et al. (2024) utilized hyperspectral data acquired via UAVs and applied the Successive Projections Algorithm (SPA) to select optimal bands. By developing four distinct classification models (SVM, RF, 1D-CNN, and 3D-CNN), their research proved that identifying barnyardgrass within intricate paddy field settings is highly viable. Similarly, Bazrafkan et al. (2024) utilized UAV multispectral data and extracted NDVI, plant area, and aspect ratio features, while integrating SMOTE-ENN for data balancing and PCA-based recursive feature elimination for variable selection, thereby achieving robust weed detection in maize fields. In addition, Yang (2024) performed K-means clustering on the *a*-channel of the Lab color space and extracted color and mLBP texture features to develop a K-SRC classification model combining KNN and OMP, which effectively distinguished nine common weed species in natural vegetable field environments.

Despite these advances, traditional machine learning methods still suffer from several inherent limitations. Most notably, they rely heavily on manual feature engineering, which requires substantial domain expertise and may fail to capture subtle or complex patterns in high-dimensional data, particularly hyperspectral imagery. In addition, their generalization ability is often limited in diverse agricultural environments, where variations in illumination, soil background, occlusion, and weed growth stage can substantially degrade performance. Classifiers such as SVM and RF may also encounter overfitting or underfitting when trained on imbalanced datasets commonly collected in field surveys, thereby requiring additional preprocessing strategies such as data augmentation or class rebalancing.

### 2.2 Deep learning approaches for crop weed identification

The emergence of deep learning has fundamentally transformed crop weed identification, shifting the paradigm from handcrafted feature engineering to automated hierarchical feature learning. Deep neural networks are capable of directly learning multi-level abstract representations from raw images, thereby significantly improving adaptability while reducing dependence on manual intervention (Zhang et al., 2025a). Compared with traditional approaches, deep learning models generally exhibit stronger robustness to illumination variation, occlusion, and complex backgrounds, making them well suited to end-to-end learning in challenging agricultural environments (Liu et al., 2024c).

#### 2.2.1 Deep learning-based classification and segmentation methods for crop weed identification

Classification-based methods aim to assign input images or local patches to predefined categories, such as crop species, weed species, or background classes (Tsai et al., 2022). These approaches typically employ architectures such as convolutional neural networks (CNNs), ResNet, and Vision Transformers to automatically learn discriminative representations. They are particularly effective in large-scale agricultural monitoring scenarios, where multimodal inputs can be processed to achieve high recognition accuracy under variable illumination conditions and across different growth stages (Yuan et al., 2025).

Concurrently, approaches based on segmentation offer detailed delineation of weeds and crops at the pixel or instance scale. Architectures designed for semantic segmentation, including U-Net (Ronneberger et al., 2015) and DeepLabv3+ (Chen et al., 2018), produce high-resolution masks to distinguish crops from weeds. In contrast, instance segmentation models like Mask R-CNN (He et al., 2018) facilitate the accurate extraction of the contours of single plants. As a prominent case, the WeedSense system (Sarker et al., 2025) employs a multi-task learning paradigm to jointly handle density mapping, plant height estimation, and weed segmentation. This approach surpasses various baseline models across different datasets, particularly regarding mIoU and F1-score metrics. In another study, a hybrid deep learning framework used segmentation masks to suppress background interference, enabling accurate estimation of weed density and growth rate in complex scenes (Mishra et al., 2025). Likewise, the AWRC-DLMLO framework (Gopalakrishnan et al., 2025) combines an attention-enhanced segmentation module with a lightweight classifier and achieves competitive real-time recognition performance.

Although classification and segmentation methods improve interpretability and may reduce computational overhead compared with more complex detection frameworks, they often require separate stages for localization and identification. Such multi-step pipelines may limit efficiency and deployment flexibility in precision agriculture. To satisfy the practical requirements of real-time field applications, object detection methods have attracted increasing attention because they unify localization and recognition within a single framework.

#### 2.2.2 Deep learning-based object detection methods for crop weed identification

By combining classification and localization within a single framework, deep learning-driven object detection algorithms facilitate the real-time extraction of bounding boxes and class labels for weed recognition tasks (Li et al., 2024b). Leading networks, including DETR-derived models, Faster R-CNN, and the YOLO family, have proven highly effective at managing complicated plant arrangements and multiscale targets in farming environments (Chen et al., 2025b). Compared with classification and segmentation methods, object detection is more suitable for real-time weed management because it simultaneously provides category information and spatial locations of weeds. However, agricultural detection differs from general object detection because weed targets are often small, irregularly distributed, partially occluded, and highly similar to crop leaves. Therefore, successful weed detectors must balance global contextual perception, fine-grained local-detail preservation, and efficient feature fusion across multiple scales.

To illustrate, Lu et al. (2025) successfully identified twelve distinct cotton field weeds with high precision by modifying the YOLOv8 architecture. They achieved this by integrating the LSK attention module to expand the receptive field and boost feature differentiation, alongside adopting a StarNet backbone and a lightweight shared separable convolutional BN head (LSCSBD). In a separate study, Cui et al. (2024) compiled a dataset of roughly 4,000 UAV-captured images of weeds in soybean crops. After testing VGG-19, VGG-16, ResNet-101, and ResNet-50 within a Faster R-CNN setup, they found that VGG-16 delivered the peak average precision (88.69%). Furthermore, Jin et al. (2025) developed the lightweight PHRF-RTDETR model to identify weeds in upland rice. Their optimized RT-DETR network substituted the GIoU loss with Focaler-WIoUv3, upgraded RepC3 to RetC3 via a RetBlock, integrated parameter-free HiLo attention into the AIFI component, and utilized a PGRNet backbone. Ultimately, these upgrades secured an optimal trade-off between processing speed and accuracy under intricate field conditions. These studies demonstrate that directly applying general-purpose detectors to agricultural scenes is often insufficient. Instead, task-specific structural improvements are usually required to address multi-scale target variation, dense occlusion, complex backgrounds, and the need for lightweight deployment.

From the perspective of backbone feature extraction, multi-scale contextual modeling is one of the most critical requirements for crop weed detection. Weeds may appear at different scales due to variations in growth stage, shooting distance, plant density, and partial occlusion. Pyramid pooling and related multi-scale aggregation strategies can enlarge the receptive field and improve the perception of objects with different sizes (Zhao et al., 2017). However, directly introducing large contextual regions may also include irrelevant crop leaves, soil textures, and background clutter in the feature representation. Therefore, dense agricultural scenes require a more targeted structure that can enhance scale-aware contextual information while preserving discriminative local details. Motivated by this requirement, the Cross-Stage Multi-Scale Network (CSMN) is introduced to strengthen multi-scale contextual representation and edge-related information in the backbone. This design enables the detector to better recognize small, occluded, and scale-varying weeds under complex field conditions.

Beyond multi-scale context, local-detail representation is also essential for reliable crop–weed discrimination. In sugar beet fields, weeds and crop leaves often share similar colors, shapes, and textures, making fine-grained cues such as leaf contours, vein-like textures, boundary continuity, and subtle geometric differences particularly important for accurate localization and classification. Existing atrous-convolution and multi-branch structures can enhance local feature extraction, but large dilation rates may weaken small object boundaries, while complex hierarchical structures may increase computational overhead. To address these limitations, the Cross-Stage Local-Detail Module (CSLM) is designed to enhance high-frequency texture and boundary cues while maintaining semantic stability and real-time efficiency. By incorporating CSLM into the neck, the detector can better preserve local discriminative details after backbone feature extraction, thereby reducing missed detections and inaccurate localization caused by crop–weed visual similarity and occlusion.

Feature resampling is another important factor affecting detection performance, because multi-scale features are repeatedly upsampled and downsampled during feature fusion. Conventional interpolation, pooling, strided convolution, and transpose convolution can efficiently change feature resolution, but they may also introduce semantic inconsistency, spatial misalignment, or boundary distortion during cross-scale propagation. For weed detection in dense fields, this problem is especially harmful because small targets and weak boundaries are easily disturbed during feature transformation. Therefore, the Global Attention-Gated Dual-Path Upsampler and Downsampler (GAGDU and GAGDD) are introduced to improve cross-scale feature transition. By combining dual-path resampling with global attention-guided channel gating, these modules help preserve both semantic continuity and spatial structure, leading to more stable representations for small, overlapping, and irregularly distributed weeds.

Even with such progress, current detection pipelines face notable constraints when applied to agriculture. Because of their single-stage structure, YOLO variants excel in real-time processing; however, relying on non-maximum suppression (NMS) and preset anchors restricts their efficacy in heavily occluded areas with irregular weed-crop overlap (Ren et al., 2025; Liu et al., 2025b). Conversely, while two-stage models like Faster R-CNN yield superior localization accuracy, their reliability frequently drops when exposed to varied lighting and heterogeneous field backgrounds (Yu et al., 2024). Frameworks built on DETR (e.g., RT-DETR) overcome these anchor and NMS dependencies by utilizing transformer-driven global attention, concurrently capturing multi-scale contextual links and long-range dependencies (Zheng et al., 2025). Driven by these inherent benefits, this paper selects a DETR-based foundation as its primary detection structure, aiming to elevate the resilience and precision of weed identification amidst complex agricultural settings.

## 3 Materials and methods

### 3.1 Dataset collection, pre-processing and annotation

**Figure 2:**
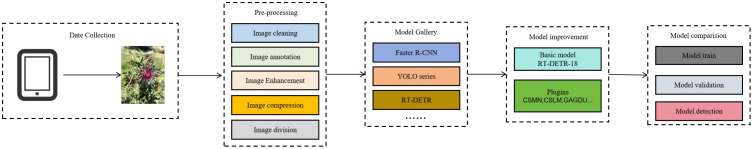
Proposed research framework for data collection, pre-processing, and training.

As depicted in **Figure 2**, we construct an end-to-end pipeline for the object detection training phase, breaking down the overall procedure into distinct modular components. The provided block diagram maps out the full operational workflow—spanning from initial data preprocessing through to final model evaluation—thereby serving as an intuitive guide to clarify every distinct phase of the training methodology.

#### 3.1.1 Collection of a nine-class sugar beet weed image

**Figure 3:**
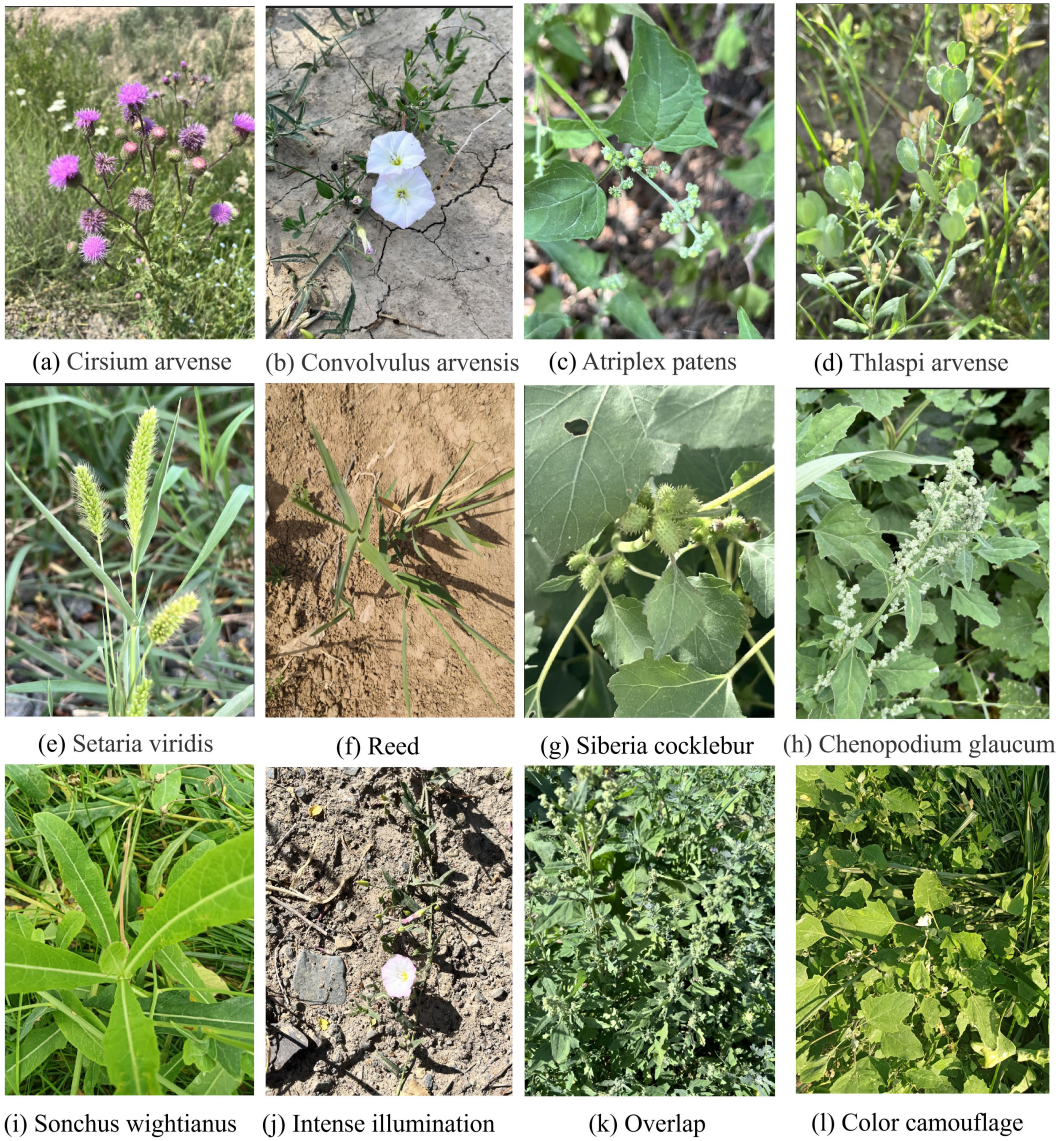
This figure shows example images from the dataset. Subfigures **(a–i)** exemplify the full set of nine categories, while **(j–l)** depict a subset of complex scenes.

The dataset analyzed in this research was assembled from sugar beet fields located in Jimsar County, Gongliu County, and Anningqu Town within China’s Xinjiang Uygur Autonomous Region. Selected for their extensive farming scales, these regions foster substantial weed growth, presenting optimal environments for documenting varied field conditions. Depicted in **Figure 3**, the collection contains nine typical weed types: Cirsium arvense, Convolvulus arvensis, Atriplex patens, Thlaspi arvense, Setaria viridis, Reed, Siberia cocklebur, Chenopodium glaucum, and Sonchus wightianus. To guarantee comprehensive and representative samples, image acquisition took place throughout the primary growth window from June to September 2024. Recording sessions spanned from 10 a.m. to 8 p.m. to capture diverse lighting situations. Furthermore, to avert motion blur induced by wind, photography was executed during stable environmental conditions. When sudden gusts occurred, repeated shots of the same target area were taken from different perspectives, ensuring a minimum of one pristine photograph was secured for accurate analysis.

Utilizing a combination of a Nikon D800, an iPhone 13, and an iPhone 15, researchers amassed 3,802 high-resolution pictures (resolutions of 4,512 × 4,512, 3,024 × 3,024, and 3,120 × 4,160 pixels). Special attention was directed toward documenting demanding agricultural scenarios. Consequently, images were captured across various distances and elevations. The corpus purposefully integrates scenes featuring thick foliage, bare soil, and intricate backgrounds, thereby amplifying its difficulty level and enhancing its utility for training models applicable to actual agricultural environments.

### 3.2 Creation of the dataset

#### 3.2.1 Data annotation

Guided by agricultural specialists, the acquired imagery was manually labeled utilizing the LabelImg tool. This effort produced a comprehensive dataset comprising nine distinct categories of sugar beet weeds, complete with corresponding.txt annotation files. To guarantee a statistically sound and balanced distribution—a critical factor for dependable model execution—the data was divided into training, validation, and testing subsets following a 7:2:1 ratio (Han et al., 2025). Specifically, the training portion supplies sufficient data for deep feature extraction, the validation segment allows for hyperparameter tuning while averting overfitting, and the test set acts as an impartial benchmark for assessing generalization on novel data. Because it successfully balances objective evaluation with learning stability, this exact proportion is a standard practice in computer vision, ultimately boosting the validity and resilience of the experimental findings (Piotr et al., 2024).

#### 3.2.2 Data enhancement

**Figure 4:**
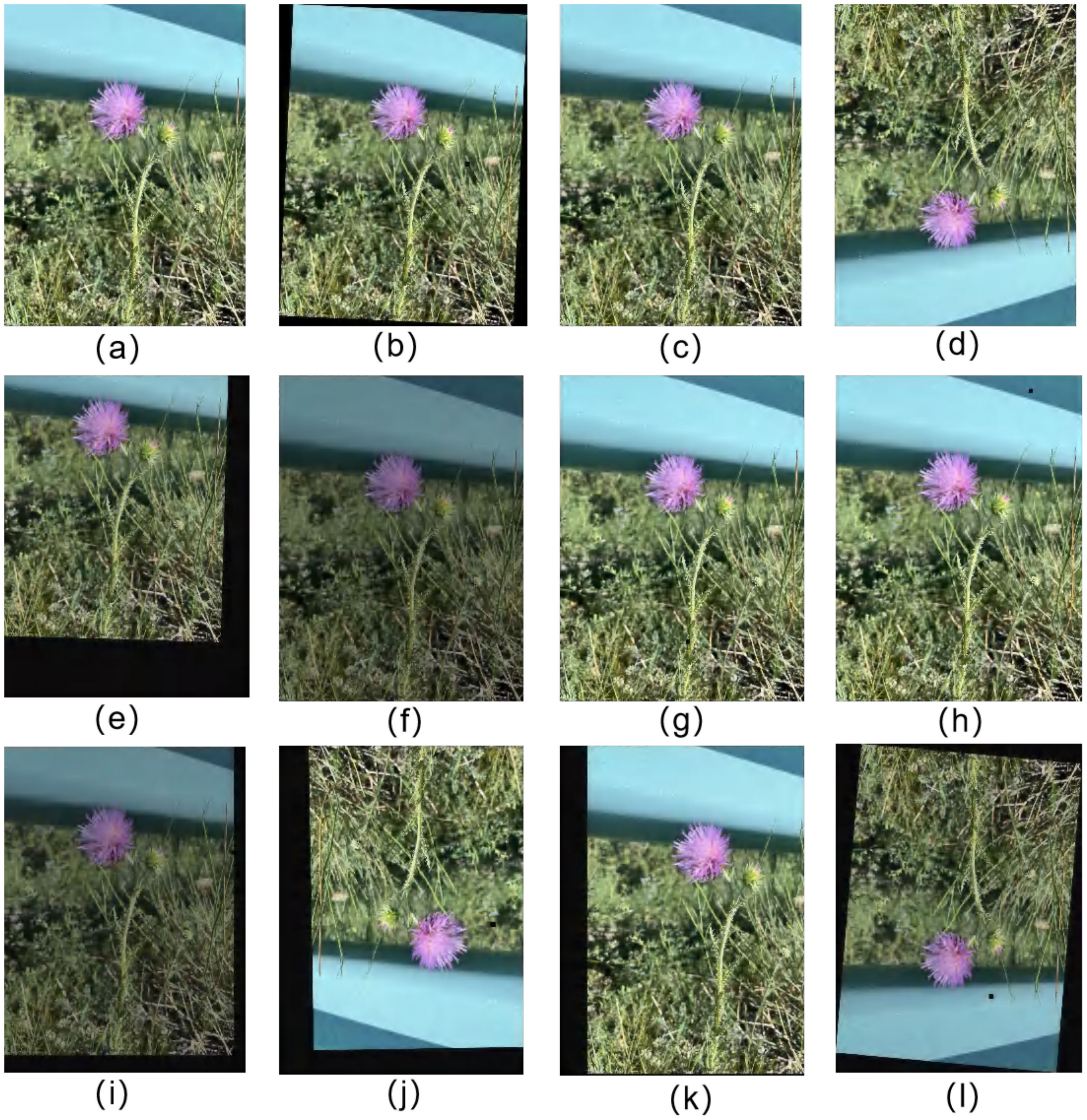
**(a)** Original image; **(b)** random rotation with affine correction and bounding box alignment; **(c)** horizontal flip; **(d)** vertical flip; **(e)** random translation within 20% of image size; **(f)** brightness adjustment with random intensity scaling (0.35–1.0); **(g)** Gaussian noise injection simulating sensor noise; **(h)** cutout regularization simulating partial occlusion; **(i)** combined brightness adjustment (0.35–1.0) and random rotation with affine correction; **(j)** vertical flip combined with 20% random translation; **(k)** Gaussian noise injection combined with brightness adjustment (0.35–1.0); **(l)** combined 20% random translation, vertical flip, and cutout regularization.

To enhance the robustness and generalization capability of the proposed detection model under complex field conditions, a comprehensive data augmentation pipeline was designed and implemented. The augmentation scheme integrates both geometric and photometric transformations while strictly maintaining the spatial consistency between images and their corresponding bounding boxes. Specifically, as illustrated in **Figure 4**, the applied operations include random rotation (± 5°) with affine bounding box correction, horizontal and vertical flipping, random translation up to 20% of the image dimensions, brightness modulation with intensity scaling between 0.35 and 1.0, Gaussian noise injection to emulate sensor-induced disturbances, and cutout regularization to simulate partial occlusions commonly encountered in natural farmland environments. Each original image was augmented five times, with at least one transformation applied during each iteration. This strategy substantially increases intra-class variability and strengthens the model’s resilience to illumination changes, occlusion patterns, and background variations, thereby improving detection stability in real-world sugar beet fields.

#### 3.2.3 Data compression

The original dataset, totaling 88.6 GB in size, posed significant challenges in terms of storage capacity, network transmission, and computational efficiency during model training. However, because weed detection relies heavily on small-target structures, fine-grained leaf textures, and boundary information, aggressive resolution reduction may potentially weaken discriminative visual cues. Therefore, in addition to reducing storage and transmission costs, we further evaluated whether image compression affected detection performance. Specifically, the spatial resolution of each image was reduced to one-quarter of its original width and height, decreasing the dataset size from 88.6 GB to 8.44 GB, corresponding to a storage reduction of approximately 90.5%. The original image resolutions were 4512 × 4512, 3024 × 3024, and 3120 × 4160 pixels, and their compressed counterparts remained approximately 1128 × 1128, 756 × 756, and 780×1040 pixels, respectively. Since these compressed images were still larger than the detector input size of 640 × 640, most target-level visual information could still be retained before network resizing. To quantitatively verify the effect of compression, CSAG-DETR was trained and evaluated on the original-resolution dataset and the compressed dataset under the same data split, input size, training strategy, and hyperparameter settings. The comparison results are reported in **Table 1**.

**Table 1:** Effect of image compression on detection performance efficiency.

Image compression	P/%	R/%	FPS	mAP@0.5/%	mAP@0.5:0.95/%	FLOPs/G	Param/M	Weight/MB
	98.1	96.7	167.3	98.5	78.9	48.1	14.3	55.1
✓	98.1	96.3	167.3	98.9	78.6	48.1	14.3	55.1

Compared with the original-resolution dataset, the compressed dataset maintained the same precision of 98.1% and the same FPS of 167.3. The recall decreased slightly from 96.7% to 96.3%, and mAP@0.5:0.95 decreased from 78.9% to 78.6%, indicating a minor loss of fine-grained localization information after compression. Nevertheless, mAP@0.5 increased from 98.5% to 98.9%, suggesting that the overall detection accuracy remained stable. Since FLOPs, parameter count, and model weight are determined by the network architecture, these efficiency indicators remained unchanged before and after compression. Overall, these results show that the adopted compression strategy substantially reduced the dataset size while causing only a very limited performance fluctuation. Although slight decreases in recall and mAP@0.5:0.95 suggest that some small-target details may be affected, the magnitude of degradation was negligible relative to the large reduction in storage cost. Therefore, the compressed dataset was used in subsequent experiments as a practical trade-off between computational efficiency, storage feasibility, and reliable weed detection performance under complex field conditions. Overall, these results show that the adopted compression strategy substantially reduced the dataset size while causing only a very limited performance fluctuation. Although slight decreases in recall and mAP@0.5:0.95 suggest that some small-target details may be affected, the magnitude of degradation was negligible relative to the large reduction in storage cost. Therefore, the compressed dataset was used in subsequent experiments as a practical trade-off between computational efficiency, storage feasibility, and reliable weed detection performance under complex field conditions.

### 3.3 Transformer-based RT-DETR structure

**Figure 5:**
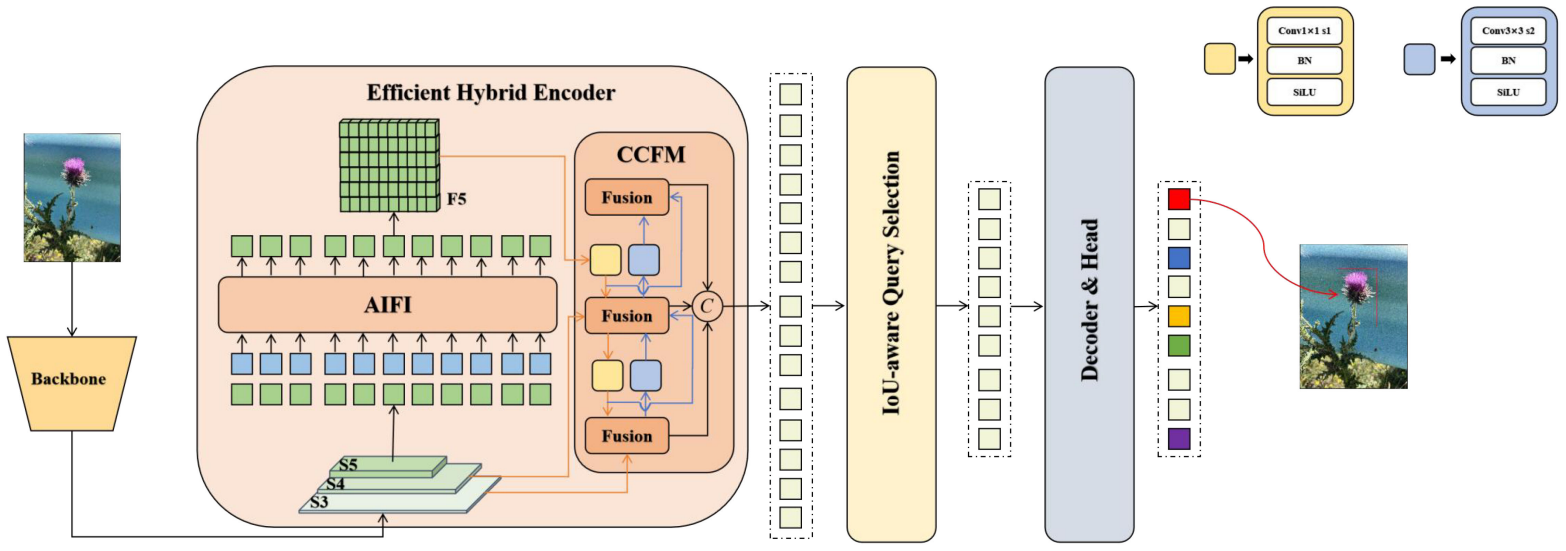
The structure of RT-DETR.

RT-DETR (Real-Time Detection Transformer) operates as a Transformer-driven architecture engineered to optimally balance inference speed with detection precision. Moving away from standard CNN paradigms—which rely heavily on non-maximum suppression (NMS), anchor generation, and customized feature pyramids—this framework processes object detection as a direct, end-to-end prediction problem. Within its structure, the encoder gathers multi-scale outputs from the backbone and maps long-range spatial connections via attention mechanisms. Concurrently, the decoder conducts query-driven reasoning to instantly yield bounding boxes and classifications, bypassing all anchor-matching steps. To optimize computational load without sacrificing accuracy, RT-DETR implements an IoU-aware query selection mechanism alongside a streamlined hybrid encoder. Depicted in **Figure 5**, the system is built upon three primary components: a convolutional backbone for initial feature extraction, a Transformer encoder handling multi-scale interactions, and a simplified decoder for parallel predictions. Ultimately, these features empower RT-DETR to grasp global contexts and handle intricate plant distributions at real-time speeds, establishing it as an ideal baseline for demanding agricultural applications.

### 3.4 Baseline model

To accommodate the real-world demands of identifying weeds within sugar beet crops, this research adopted RT-DETR-R18 as the foundational architecture. As demonstrated in **Table 2**, this specific model achieves the optimal trade-off between inference efficiency and detection precision when compared to other RT-DETR configurations, securing an mAP@0.5 of 97.5% alongside an mAP@0.5:0.95 of 69.0%. Furthermore, its structural footprint and computational demands remain remarkably low, characterized by just 19.9 M parameters, 57.0 GFLOPs, and a minimal storage requirement of 77.0 MB.

**Table 2:** Comparison of different RT-DETR variants on the BeetWeed dataset.

Model	P/%	R/%	FPS	mAP@0.5/%	mAP@0.5:0.95/%	FLOPs/G	Param/M	Weight/MB
RT-DETR-R18	94.1	94.0	196.6	97.5	69.0	57.0	19.9	77.0
RT-DETR-R34	95.9	90.4	137.9	96.2	67.6	88.8	31.1	119.9
RT-DETR-R50	96.6	90.3	92.6	96.5	66.2	129.6	42.0	163.9
RT-DETR-R101	93.4	88.5	59.9	94.3	63.5	247.1	74.7	292.9
RT-DETR-L	95.8	92.8	109.7	96.2	66.4	103.5	32.0	125.8

In this study, RT-DETR-R18 was established as the primary baseline architecture because it optimally balances deployment practicality, computational efficiency, and detection accuracy for intricate sugar beet weed recognition tasks. This decision is justified by comparisons with other variants: for instance, RT-DETR-R34 consumes more memory and processing power yet produces lower precision than R18. Meanwhile, despite demonstrating strong performance in specific metrics, heavier models (namely RT-DETR-R50, RT-DETR-R101, and RT-DETR-L) are hindered by their massive sizes and intense computational overhead, making them largely impractical for resource-limited agricultural applications.

### 3.5 CSAG-DETR network model

Accurately identifying weeds among sugar beet crops is notoriously difficult, primarily because of their stark visual resemblance, compounded by fluctuating lighting, overlapping leaves, and thick foliage. To be viable for precision farming, a system must not only recognize plants accurately but also operate within the tight computational limits of real-time, edge-device hardware. Thus, the ideal framework must perfectly synthesize processing efficiency with robust feature extraction. Recognizing RT-DETR as a leading architecture that expertly navigates the accuracy-speed compromise, we chose its R18 variant for this research due to its exceptional blend of implementability, low complexity, and detection capability.

**Figure 6:**
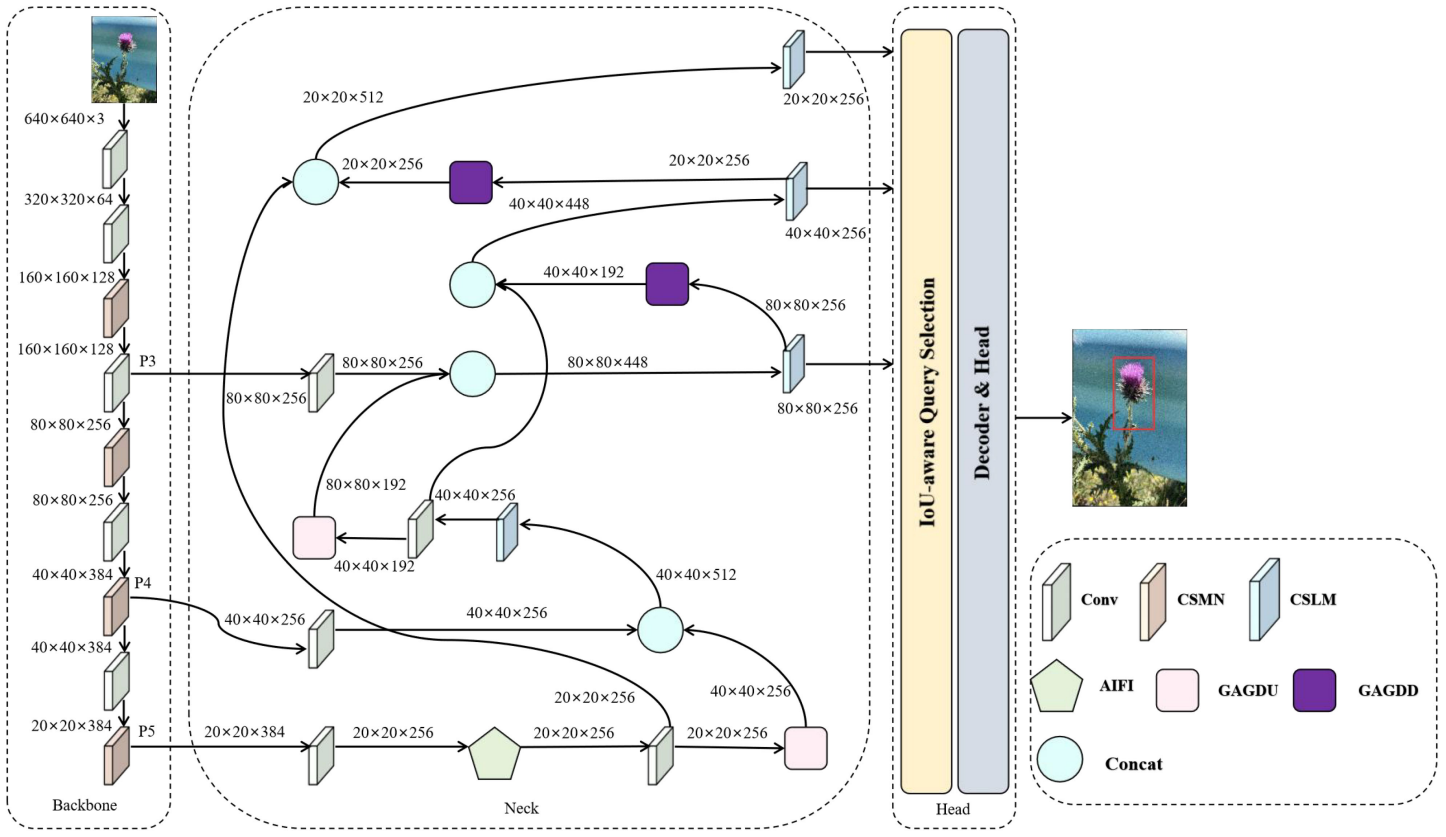
The overall architecture of the proposed CSAG-DETR model.

Despite its baseline strengths, the standard RT-DETR-R18 struggles with the specific nuances of sugar beet environments. Background noise, densely packed plants, and nearly identical textural features impede the network’s capacity to pass multi-scale features steadily and retain intricate structural traits. Consequently, identifying tiny, heavily overlapped, or camouflaged weed specimens becomes problematic. Overcoming these hurdles is the primary goal of our proposed CSAG-DETR (Cross-Stage Attention-Gated DETR). Tailored expressly for sugar beet cultivation, this efficient, real-time framework’s complete structural blueprint is depicted in **Figure 6**.

First, a Cross-Stage Multi-Scale Network (CSMN) is introduced into the backbone to strengthen hierarchical feature interaction and multi-scale contextual representation, thereby improving the extraction of fine-grained textures and edge information from visually similar vegetation targets. Second, a Cross Stage Local-Detail Module (CSLM) is incorporated into the neck to enhance local-detail modeling and preserve high-frequency texture and boundary cues that are easily weakened during repeated feature abstraction. Finally, the Global Attention-Gated Dual-Path Upsampler (GAGDU) and Downsampler (GAGDD) are introduced to replace conventional feature resampling operations, enabling more stable cross-scale feature transition through globally guided channel gating and dual-path structural compensation. By integrating these modules into a unified detection framework, CSAG-DETR achieves more robust, accurate, and efficient weed detection in sugar beet fields under complex real-world conditions.

#### 3.5.1 Cross-stage multi-scale network

**Figure 7:**
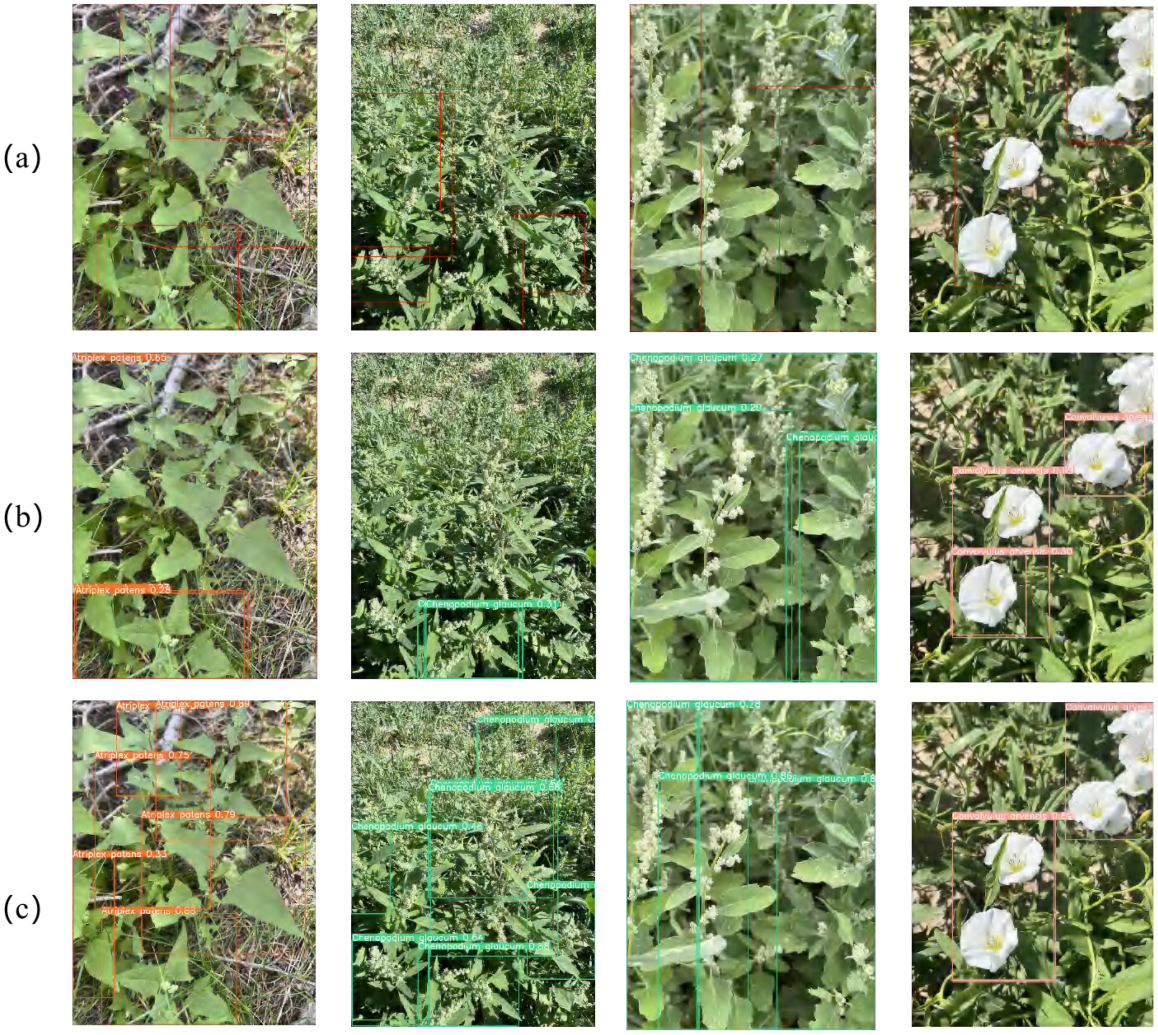
**(a)** Ground truth examples. **(b)** Detection results using RT-DETR-R18 without data augmentation. **(c)** Detection results using RT-DETR-R18 with data augmentation. By comparing rows **(b)** and **(c)**, it is evident that data augmentation substantially improves detection accuracy and robustness. For instance, in the first and second columns, the augmented model successfully identifies more weed instances that were missed by the non-augmented model. In the third column, the predictions closely match the ground truth, indicating enhanced spatial precision. In the fourth column, the augmented model correctly detects the weed in the upper-right region that was previously overlooked. Overall, the augmentation strategy leads to more complete and accurate recognition of diverse weed species under complex field conditions.

**Figure 8:**
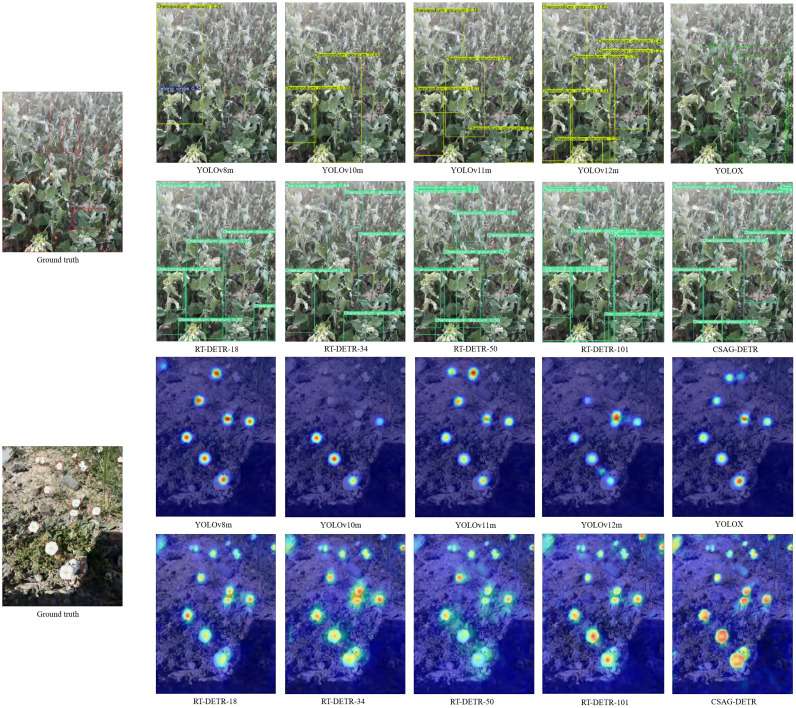
Examples of detection results and corresponding heatmap comparisons of different algorithms. The visualization results indicate that various state-of-the-art algorithms exhibit significant differences in sugar beet weed detection performance under complex farmland environments. Compared with the ground-truth results (leftmost column), the proposed CSAG-DETR shows better localization accuracy. Although CSAG-DETR has a few missed detections (such as in the lower right corner of the first image), its overall detection performance is still superior to other advanced algorithms. It is worth noting that the CNN-based YOLO series show insufficient attention under strong illumination, occlusion, and small-object detection (as in the upper right corner of the second heatmap), while the Transformer-based DETR model performs moderately and occasionally exhibits attention deviation from weed targets. In contrast, the detection boxes of CSAG-DETR have higher spatial alignment with the ground-truth annotations. These visual results further verify the model’s dual optimization capability in reducing both missed detections and false positives under complex environments.

**Figure 9:**
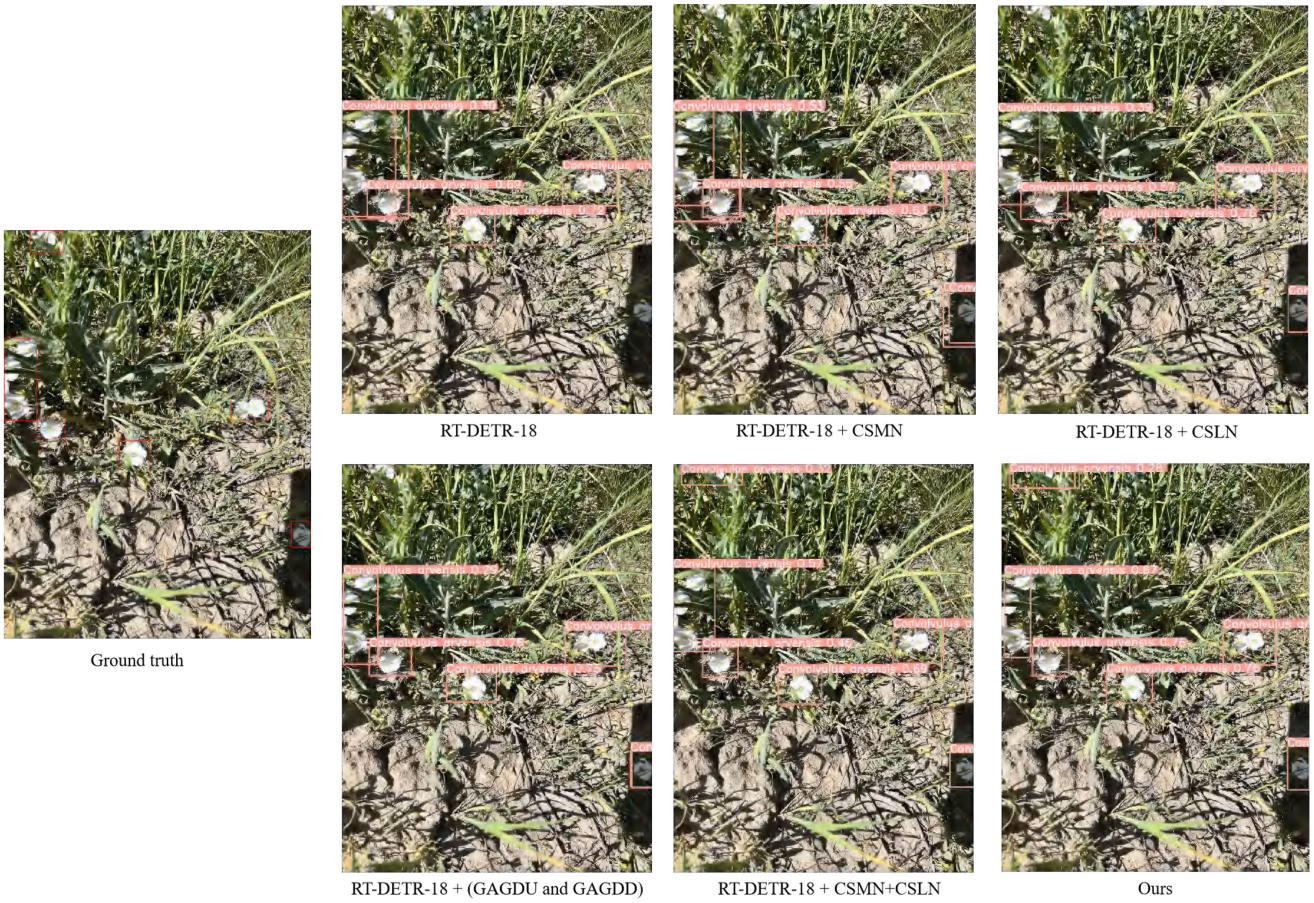
Visual comparison of detection results obtained with different module combinations.

As illustrated in **Figures 7**–**9**, weeds in real sugar beet fields often appear as small, irregular, and partially occluded targets under strong illumination variation and cluttered backgrounds. These characteristics make weed detection particularly challenging and frequently lead to missed detections and false positives. Although the original RT-DETR-R18 backbone provides effective hierarchical semantic abstraction, it is relatively insensitive to fine-grained edges, local textures, and geometric structures, which are essential for distinguishing overlapping crops and weeds (Chen et al., 2025a). In addition, its multi-scale representation mainly emphasizes semantic abstraction and lacks task-oriented optimization for agricultural weed detection, resulting in suboptimal feature fusion under complex field interference.

Moreover, real farmland images usually contain abundant background noise, such as soil textures, plant debris, specular reflections, and shadows. During repeated downsampling and high-level semantic abstraction, weak but discriminative local cues can be suppressed, making small weed structures more difficult to preserve. This degradation limits both detection accuracy and robustness. Therefore, enhancing background suppression, detail preservation, and cross-scale feature interaction is crucial for reliable weed detection in complex field environments.

**Figure 10:**
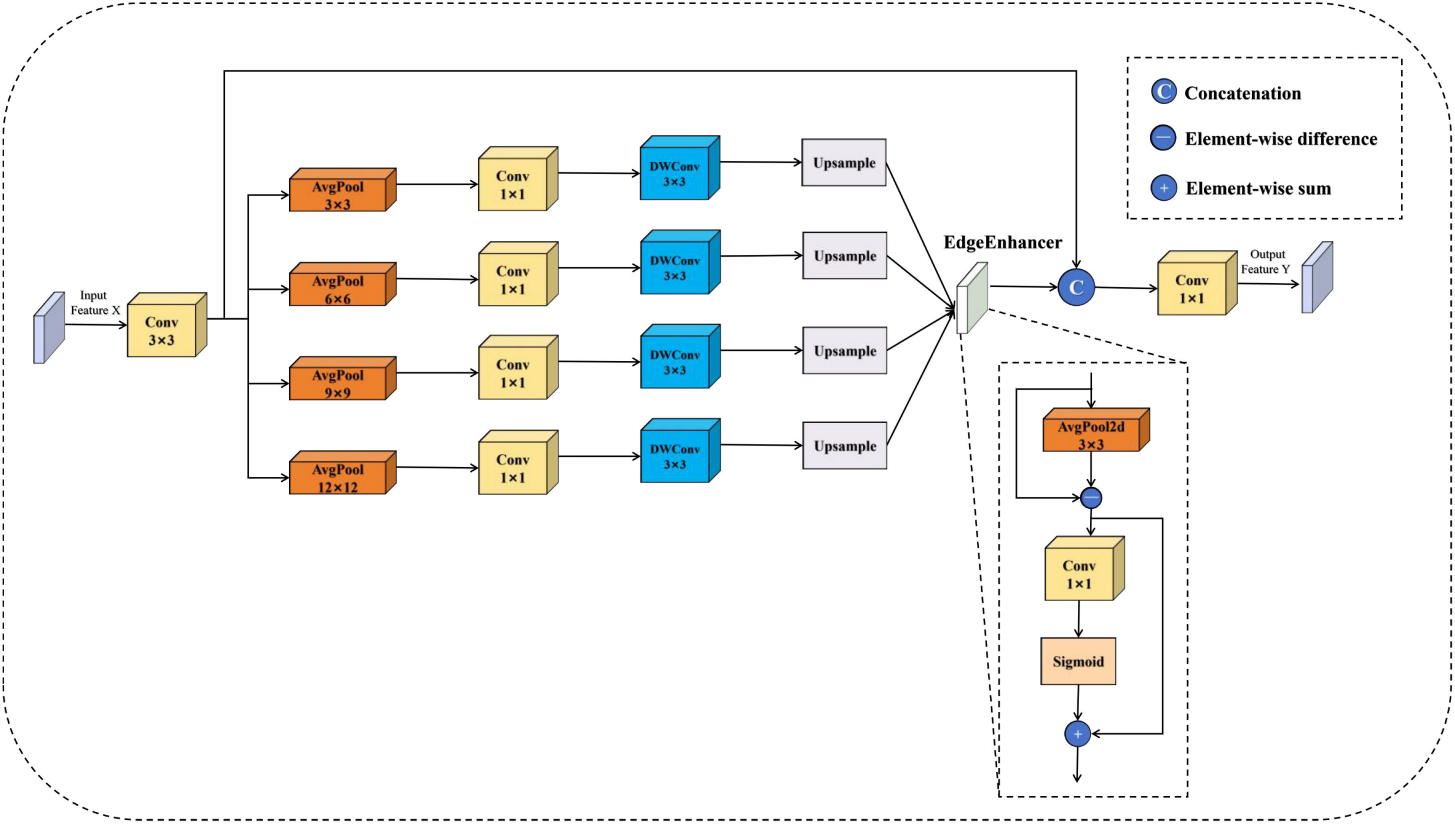
The structure of CSMN.

To tackle the aforementioned challenges, this paper proposes a Cross-Stage Multi-Scale Network (CSMN). The overall architecture of CSMN is illustrated in **Figure 10**, and the detailed layer configurations are listed in **Table 3**. Building upon existing multi-scale and cross-stage feature representation modules, CSMN selectively absorbs valuable design paradigms and is reconstructed and optimized to meet the unique requirements of dense weed detection in sugar beet fields. Firstly, CSMN inherits the multi-scale context aggregation concept of the Pyramid Pooling Module (PPM) (Zhao et al., 2017). Unlike conventional schemes that only introduce pooled context at high-level semantic stages, the proposed network embeds adaptive pooling branches into multiple stages of the backbone, and adopts lightweight depth-wise convolutions to refine each pooled feature stream. This architecture can better retain small weed targets, faint object boundaries and subtle leaf textures, preventing such information from being continuously degraded during repeated downsampling operations. Secondly, another distinction between CSMN and standard pyramid pooling modules lies in the lightweight depth-wise convolution appended after each pooling branch. This operation refines pooled features via local spatial filtering, enabling the module to preserve local texture and boundary information while expanding the receptive field. As a result, CSMN is no longer limited to coarse context aggregation and achieves better adaptability to field images disturbed by soil textures, shadows, overlapping leaves, plant residues and other noise. Thirdly, CSMN draws on the feature reuse principle of CSP-style architectures (Wang et al., 2020), yet they possess fundamentally different design objectives. CSP networks focus on channel splitting and gradient path partitioning, whereas the core goal of CSMN is to strengthen the propagation of detail-rich multi-scale features across all backbone stages. The module fully retains the original input features and integrates three key functions within one lightweight basic block: local detail preservation, adaptive multi-scale context encoding, and residual edge enhancement.

**Table 3:** Details of the backbone.

Layer	Input	Module	Repeat	Kernel	Output
0	640^2^×3	Conv	1	3	64
1	320^2^×64	Conv	1	3	128
2	160^2^×128	CSMN	1	3	128
3	160^2^×128	Conv	1	3	256
4	80^2^×256	CSMN	1	3	256
5	80^2^×256	Conv	1	3	384
6	40^2^×384	CSMN	1	3	384
7	40^2^×384	Conv	1	3	384
8	20^2^×384	CSMN	1	3	384

Specifically, given an input feature map $X\in {\mathbb{R}}^{C\times H\times W}$, CSMN first applies a local convolution branch to preserve spatial details, as formulated in Equation (1):

(1) $$F_{\text{local}}={\text{Conv}}_{3\times 3}(X).$$

In parallel, a set of multi-scale context branches is constructed using adaptive average pooling with output sizes B={3,6,9,12}. For each scale $b_{i}\in B,$ the pooled feature is processed by a 1 × 1 convolution for channel reduction, followed by a depth-wise 3 × 3 convolution for lightweight local refinement, and then upsampled to the original spatial resolution as described in Equation (2):

(2) $$F_{i}=\text{Up }({\text{DWConv}}_{3\times 3}({\text{Conv}}_{1\times 1}({\text{AdaptiveAvgPool}}_{b_{i}}(X)))),\;b_{i}\in B.$$

The resulting multi-scale features are aggregated as described in Equation (3).

(3) $$F_{\text{ms}}=\text{Concat}(F_{1},F_{2},F_{3},F_{4}).$$

To emphasize discriminative boundary information, the aggregated multi-scale feature is further refined by an edge-enhancement operation. A local average-pooled response is first used to approximate lowfrequency background information, and the high-frequency residual is then extracted by element-wise subtraction, as shown in Equation (4):

(4) $$E=F_{\text{ms}}-{\text{AvgPool}}_{3\times 3}(F_{\text{ms}}).$$

Next, a lightweight gating mask is generated through a 1×1 convolution followed by a sigmoid activation, which can be expressed as Equation (5):

(5) $$M=\sigma \;({\text{Conv}}_{1\times 1}(E)).$$

The enhanced multi-scale representation is then obtained according to Equation (6).

(6) $${\hat{F}}_{\text{ms}}=F_{\text{ms}}+M,$$

which strengthens leaf contours, vein patterns, and other fine geometric structures that are easily weakened during backbone downsampling.

Finally, the enhanced multi-scale representation is concatenated with the local branch and fused by a 1 × 1 convolution to generate the output feature, as defined in Equation (7):

(7) $$Y={\text{Conv}}_{1\times 1}(\text{Concat}(F_{\text{local}},{\hat{F}}_{\text{ms}})).$$

By stacking CSMN blocks across the backbone, the network forms a cross-stage, detail-preserving feature hierarchy that enables more effective propagation of shallow structural cues into deeper semantic layers. In this way, CSMN simultaneously improves scale adaptability, edge sensitivity, and resistance to complex background interference, thereby providing more discriminative representations for weed detection in sugar beet fields.

#### 3.5.2 Cross-stage local-detail module

**Figure 11:**
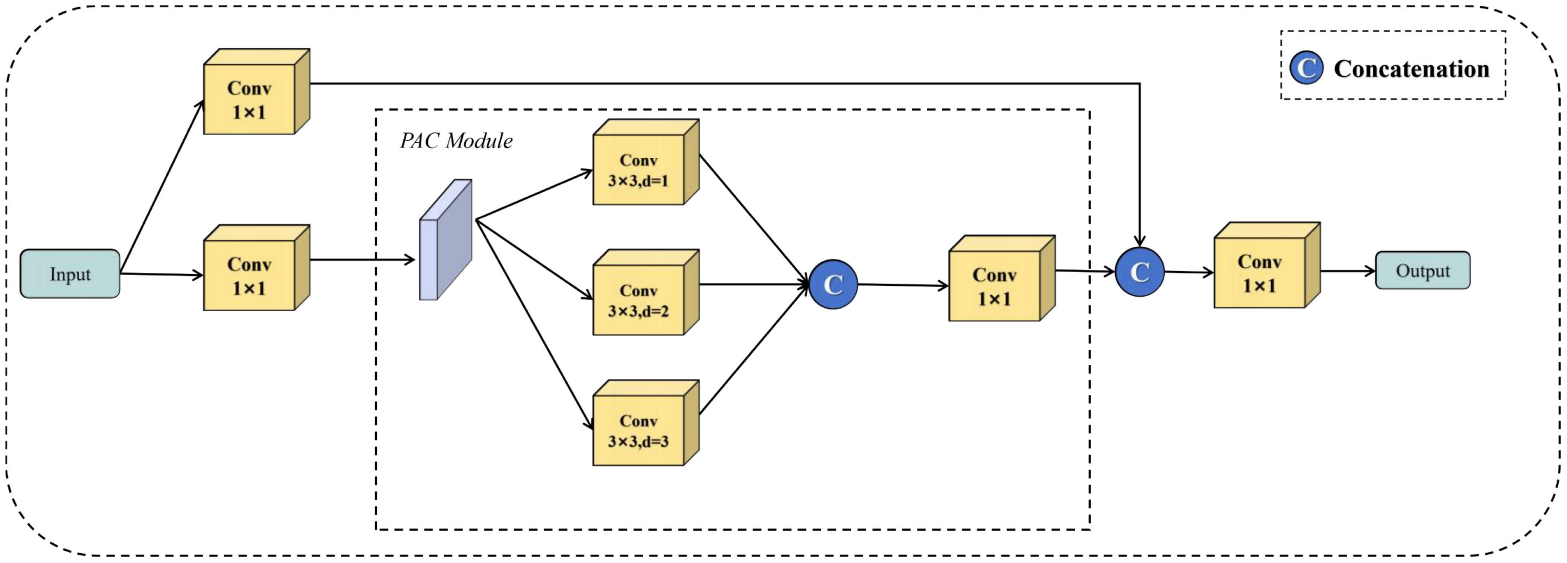
The structure of CSLM.

Although CSMN strengthens multi-scale contextual modeling and hierarchical feature interaction, its emphasis remains mainly on semantic enhancement. In dense sugar beet fields, however, reliable crop–weed discrimination also depends heavily on fine-grained local cues, such as leaf boundaries, vein-like textures, and subtle geometric differences among visually similar plants. These cues are often weakened during repeated downsampling and feature abstraction, especially under occlusion, cluttered backgrounds, and illumination variation. To further improve the representation of local structural details, we introduce the Cross-Stage Local-Detail Module (CSLM), as illustrated in **Figure 11**.

The design of CSLM is inspired by several existing multi-scale feature extraction paradigms, while being reformulated for dense agricultural weed detection. First, CSLM inherits the multi-scale receptive-field modeling idea of Atrous Spatial Pyramid Pooling (ASPP) (Chen et al., 2019), but uses only small dilation rates to focus on local texture variation and boundary continuity rather than broad semantic context. This modification reduces the gridding artifacts commonly associated with large-rate atrous convolutions and is more suitable for small and partially occluded weed targets. Second, CSLM adopts the branch-wise feature extraction strategy of Inception-style modules (Szegedy et al., 2015), but replaces generic heterogeneous branches with a task-oriented dual-path design consisting of a semantic-preservation branch and a detail enhancement branch. Third, following the lightweight multi-scale representation philosophy of Res2Net (Gao et al., 2019), CSLM employs simple parallel aggregation rather than hierarchical split-transform-fuse operations, thereby improving efficiency while avoiding unnecessary structural complexity.

As shown in **Figure 11**, the input feature map $x\in {\mathbb{R}}^{C\times H\times W}\;$ is first projected into two parallel branches through separate 1 × 1 convolutions. One branch serves as a semantic-preservation path to retain stable feature information from the original representation, while the other branch is sent to the PAC module for local-detail enhancement. Within the PAC module, the reduced feature is processed by three parallel 3 × 3 atrous convolutions with dilation rates *d* = 1, *d* = 2, and *d* = 3, respectively, so as to capture complementary local patterns at different receptive-field scales. The outputs of these branches are concatenated and fused by a 1 × 1 convolution. The fused PAC feature is then concatenated with the semantic-preservation branch and compressed by a final 1 × 1 convolution to produce the output feature. Through this design, CSLM enhances high-frequency textures and boundary cues while preserving semantic stability and computational efficiency.

Formally, given the input feature *x*, the semantic-preservation branch is defined as Equation (8).

(8) $$x_{s}={\text{Conv}}_{1\times 1}^{\left(s\right)}(x),$$

and the feature fed into the PAC branch is expressed in Equation (9).

(9) $$x_{p}={\text{Conv}}_{1\times 1}^{\left(p\right)}(x).$$

The PAC branch applies three parallel atrous convolutions with different dilation rates, which can be formulated as Equation (10):

(10) $$f_{1}={\text{Conv}}_{3\times 3}^{d=1}(x_{p}),\;f_{2}={\text{Conv}}_{3\times 3}^{d=2}(x_{p}),\;f_{3}={\text{Conv}}_{3\times 3}^{d=3}(x_{p}).$$

These multi-scale local-detail features are concatenated and fused according to Equation (11).

(11) $$f_{\text{pac}}={\text{Conv}}_{1\times 1}\;(\text{Concat}(f_{1},f_{2},f_{3})).$$

Finally, the fused PAC representation is combined with the semantic-preservation branch to generate the output feature, as defined in Equation (12):

(12) $$y={\text{Conv}}_{1\times 1}\;(\text{Concat}(f_{\text{pac}},x_{s})).$$

By explicitly decoupling local-detail enhancement from semantic preservation, CSLM provides more discriminative representations for small, overlapping, and visually similar weeds, while maintaining the lightweight property required for real-time detection in complex sugar beet field environments.

#### 3.5.3 Global attention-gated dual-path upsampler and downsampler

**Figure 12:**
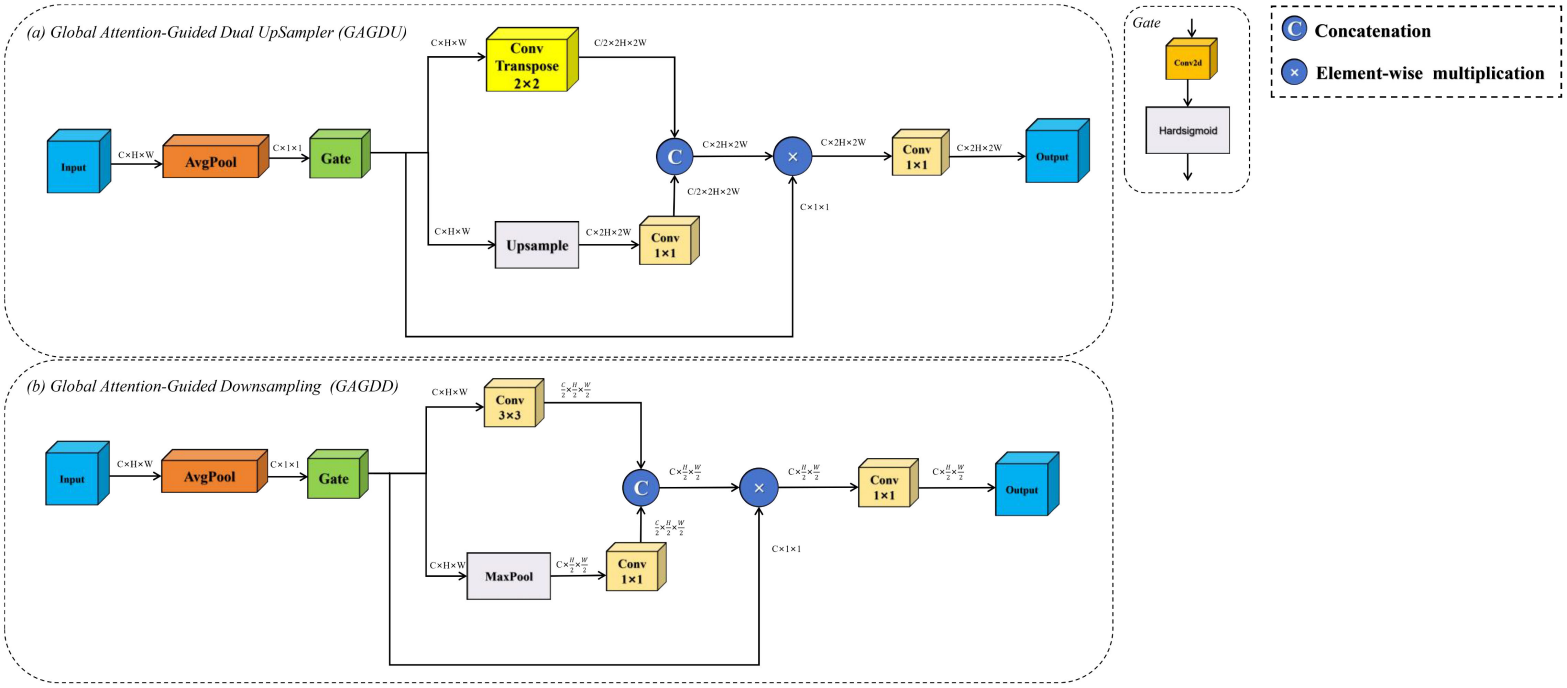
**(a)** The structure of GAGDU **(b)** The structure of GAGDD.

Although CSMN and CSLM improve contextual modeling and local-detail representation, cross-scale feature transformation in the neck may still introduce semantic inconsistency and structural distortion, especially in dense sugar beet fields with severe occlusion, irregular illumination, and complex background interference. Conventional upsampling and downsampling operators often emphasize either semantic abstraction or spatial continuity, but rarely balance both effectively. To address this issue, we design the Global Attention-Gated Dual-Path Upsampler (GAGDU) and Downsampler (GAGDD), as illustrated in **Figure 12**, to achieve more stable and discriminative feature transitions across resolutions.

The proposed design is inspired by both squeeze-and-excitation (SE) attention (Hu et al., 2019) and global context modeling (GCNet) (Cao et al., 2019), while being reformulated for real-time weed detection. From SE, GAGDU and GAGDD inherit the principle of channel-wise recalibration based on global statistics. However, unlike the original SE block, which is mainly used for feature refinement at a fixed spatial resolution, the proposed modules embed the channel gate directly into the resampling process, allowing the network to selectively preserve channels that are more important for semantic continuity and local structure recovery. From GCNet, the proposed modules adopt the idea that local feature transformation should be guided by global contextual cues. Different from explicit non-local context modeling, however, GAGDU and GAGDD use a more lightweight formulation in which the globally aggregated descriptor is directly converted into a channel gating signal. This design preserves the benefit of global-context-aware modulation while avoiding the computational burden of more complex attention operations.

Based on this design, feature resampling is decomposed into two complementary paths: a semantic main path and a detail compensation path. In GAGDU, the transpose-convolution branch emphasizes learnable semantic reconstruction, whereas the interpolation branch preserves spatial continuity and boundary details during resolution enlargement. In GAGDD, the strided-convolution branch focuses on semantic compression, while the pooling branch retains relatively stable structural information during resolution reduction. The outputs of the two paths are concatenated and modulated by a global attention gate, and then fused by a final 1 × 1 convolution. In this way, the proposed modules improve semantic alignment and spatial fidelity simultaneously during cross-scale feature propagation.

Formally, let $\text{x}\in {\mathbb{R}}^{B\times C\times H\times W}$ denote the input feature map, where *B*, *C*, *H*, and *W* represent the batch size, channel number, height, and width, respectively. A global channel descriptor is first obtained by global average pooling, as formulated in Equation (13):

(13) $$p=\text{GAP }(x),\;p\in {\mathbb{R}}^{B\times C\times 1\times 1}.$$

This descriptor is then transformed into a channel-wise gating signal through a lightweight 1 × 1 convolution followed by a hard-sigmoid activation, as described in Equation (14):

(14) $$g=\sigma_{h}\;(\underset{1\times 1}{\text{Conv}}(p)),\;g\in {\mathbb{R}}^{B\times C\times 1\times 1},$$

where the hard-sigmoid activation function is defined as Equation (15).

(15) $$\sigma_{h}(z)=\;(\text{max }(z/3+1/2,\;0),\;1).$$

For GAGDU, the input feature is processed by two parallel upsampling paths. The first path adopts a transpose convolution for learnable semantic reconstruction, as expressed in Equation (16):

(16) $$u_{1}={\text{ConvTranspose}}_{2\times 2}^{s=2}(x),\;u_{1}\in {\mathbb{R}}^{B\times \frac{C}{2}\times 2H\times 2W}.$$

The second path uses interpolation followed by a 1 × 1 convolution to preserve spatial continuity, as described in Equation (17):

(17) $$u_{2}=\underset{1\times 1}{\text{Conv}}\;(\text{Up }(x)),\;u_{2}\in {\mathbb{R}}^{B\times \frac{C}{2}\times 2H\times 2W}.$$

The two branches are concatenated and modulated by the global gate, as formulated in Equation (18):

(18) $$u=\text{Concat }(u_{1},u_{2}),\;\hat{u}=u⊙g,$$

where ⊙ denotes element-wise multiplication with channel-wise broadcasting. The final upsampled output is obtained according to Equation (19).


(19) $$y_{\text{up}}={\text{Conv}}_{1\times 1}(\hat{u}),\;y_{\text{up}}\in {\mathbb{R}}^{B\times C\times 2H\times 2W}.$$

Similarly, for GAGDD, two parallel downsampling paths are constructed. The first path uses a 3 × 3 strided convolution for semantic compression, as described in Equation (20):

(20) $$d_{1}={\text{Conv}}_{3\times 3}^{s=2}(x),\;d_{1}\in {\mathbb{R}}^{B\times \frac{C}{2}\times \frac{H}{2}\times \frac{W}{2}}.$$

The second path adopts max pooling followed by a 1 × 1 convolution to preserve structural information, as expressed in Equation (21):

(21) $$d_{2}=\underset{1\times 1}{\text{Conv}}\;({\text{MaxPool}}_{2\times 2}^{s=2}(x)),\;d_{2}\in {\mathbb{R}}^{B\times \frac{C}{2}\times \frac{H}{2}\times \frac{W}{2}}.$$

After branch concatenation and attention-guided modulation, the downsampled output is computed as defined in Equation (22). The final downsampled output is then generated according to Equation (23).

(22) $$d=\text{Concat }(d_{1},d_{2}),\;\hat{d}=d⊙g,$$

(23) $$y_{\text{down}}={\text{Conv}}_{1\times 1}(\hat{d}),\;y_{\text{down}}\in {\mathbb{R}}^{B\times C\times \frac{H}{2}\times \frac{W}{2}}.$$

Through this globally guided dual-path design, GAGDU and GAGDD reduce resampling-induced artifacts while dynamically emphasizing channels that are more informative for semantic preservation and structural recovery. Consequently, they provide more reliable multi-scale feature propagation for dense and visually similar weed targets, thereby improving the overall robustness of CSAG-DETR in real-time sugar beet weed detection.

## 4 Experimental results and analysis

To guarantee the reproducibility of our experimental results, the CSAG-DETR architecture was developed and trained within a strictly controlled environment. The underlying platform operated on a 64-bit Ubuntu 20.04 OS, powered by an Intel(R) Gold 6326 CPU (2.90 GHz) alongside an NVIDIA A30 GPU. Software wise, we relied on Python 3.8.20, Anaconda, and the VSCode IDE, implementing the models via the PyTorch 2.4.1 framework, supported by cuDNN 9.1.0 and CUDA 12.1. During the training phase, the network processed input images sized at 640×640×3 across 100 epochs with a batch size of 8. Optimization was handled by the AdamW algorithm, strategically configured with a learning rate of 0.0001, a weight decay of 0.0001, and a momentum of 0.9.

### 4.1 Evaluation indicators

The performance of the proposed model is quantitatively assessed using several widely adopted metrics, including Precision (P, %), Recall (R, %), mean Average Precision at an IoU threshold of 0.5 (mAP@0.5, %), and the averaged mAP across IoU thresholds from 0.5 to 0.95 with a step size of 0.05 (mAP@0.5:0.95, %). In addition, model complexity and computational efficiency are evaluated using the number of parameters (Param, M) and floating-point operations (GFLOPs, G). Together, these indicators provide a comprehensive evaluation of the model’s detection accuracy, robustness, and efficiency.

Precision (P) and Recall (R) are defined in Equations (24) and (25):

(24) $$P=\frac{TP}{TP+FP},$$

(25) $$R=\frac{TP}{TP+FN},$$

where *TP*, *FP*, and *FN* denote the numbers of true positives, false positives, and false negatives, respectively. The Average Precision (AP) is calculated as the area under the precision–recall (PR) curve, as formulated in Equation (26).

The mean Average Precision (mAP) is computed as the area under the precision–recall (PR) curve, as formulated in Equation (27):

(26) $$AP=\int_{0}^{1}P(R)\;dR,$$

(27) $$mAP=\frac{1}{N}\sum_{i=1}^{N}AP_{i},$$

where *P*(*R*) denotes precision as a function of recall, and *N* represents the total number of object categories. The metric *mAP*@0.5 corresponds to the average precision calculated at an IoU threshold of 0.5, while *mAP*@0.5:0.95 follows the COCO evaluation protocol by averaging AP over ten IoU thresholds from 0.5 to 0.95.

Finally, the number of parameters (Param, M) measures model complexity, whereas floating-point operations (GFLOPs, G) indicate computational cost, both of which are essential for evaluating deployment feasibility in real-world agricultural scenarios.

### 4.2 Independent contribution analysis of data augmentation operations

To further analyze the independent contribution of each augmentation operation, we conducted an operation-level ablation study. In each experiment, only one augmentation operation was applied during training, while the model architecture, data split, input size, optimizer, training epochs, and other hyperparameters were kept unchanged. This setting allows us to evaluate whether the performance improvement mainly comes from a single dominant operation or from the complementary effects of multiple transformations. The detailed results are shown in **Table 4**.

**Table 4:** Independent contribution of different data augmentation operations.

Augmentation setting	P/%	R/%	mAP@0.5/%	mAP@0.5:0.95/%
Without augmentation	81.2	71.3	77.5	44.2
Rotation	90.1	77.5	83.6	47.5
Horizontal flipping	86.1	75.4	82.7	46.7
Vertical flipping	85.3	76.2	81.3	45.9
Translation	90.8	76.9	84.5	50.5
Brightness adjustment	88.1	75.5	81.4	45.6
Gaussian noise	85.3	79.2	83.4	46.2
Cutout	85.7	76.4	82.4	46.1
Full augmentation pipeline	94.1	94.0	97.5	69.0

As shown in **Table 4**, all individual augmentation operations improve the detection performance compared with the setting without data augmentation, confirming that each operation contributes positively to model generalization. Among the single-operation settings, translation achieves the best overall performance, with 90.8% precision, 84.5% mAP@0.5, and 50.5% mAP@0.5:0.95. Compared with the non-augmented baseline, translation improves mAP@0.5 and mAP@0.5:0.95 by 7.0 and 6.3 percentage points, respectively. This indicates that spatial displacement is particularly important for sugar beet weed detection, because weeds in field images are irregularly distributed and may appear at different positions within the image.

Rotation also provides a clear improvement, increasing mAP@0.5 from 77.5% to 83.6% and mAP@0.5:0.95 from 44.2% to 47.5%. This suggests that rotation enhances robustness to changes in shooting angle and irregular plant posture. Horizontal and vertical flipping improve mAP@0.5:0.95 to 46.7% and 45.9%, respectively, showing that orientation variation is beneficial, although its contribution is smaller than that of translation and rotation. These results indicate that geometric transformations mainly improve the detector’s robustness to viewpoint, orientation, and target-location changes.

Photometric and noise-based augmentations contribute from another perspective. Brightness adjustment improves mAP@0.5:0.95 from 44.2% to 45.6%, indicating that illumination perturbation helps the model adapt to changes in sunlight intensity, shadows, and acquisition time. Gaussian noise achieves the highest recall among all single-operation settings, increasing recall from 71.3% to 79.2%. This suggests that noise injection helps the model detect more weed targets under sensor disturbance and complex background interference. Cutout improves mAP@0.5:0.95 to 46.1%, demonstrating that occlusion simulation is helpful for recognizing partially covered weeds in dense vegetation.

Although each individual operation improves performance, the full augmentation pipeline achieves the best results across all metrics, reaching 94.1% precision, 94.0% recall, 97.5% mAP@0.5, and 69.0% mAP@0.5:0.95. Compared with the strongest single-operation setting, namely translation, the full pipeline further improves recall by 17.1 percentage points, mAP@0.5 by 13.0 percentage points, and mAP@0.5:0.95 by 18.5 percentage points. This substantial improvement demonstrates that the benefit of data augmentation is not caused by a single transformation alone, but by the complementary effects of geometric variation, illumination perturbation, noise disturbance, and occlusion simulation. Since all augmentation operations are applied only during training, the improved detection performance does not introduce additional inference-time computational cost, model parameters, or deployment burden.

As illustrated in **Figure 7**, visual comparisons show that the augmented model not only improves quantitative metrics but also produces more stable and accurate predictions under challenging conditions, including complex illumination, partial occlusion, and heterogeneous farmland backgrounds.

### 4.3 Performance comparison among different weed categories

As shown in **Table 5**, the proposed CSAG-DETR achieves consistently strong detection performance across all weed categories, with an average precision of 98.1%, recall of 96.3%, mAP@0.5 of 98.9%, and mAP@0.5:0.95 of 78.6%. Nevertheless, noticeable performance differences can still be observed among categories. In particular, reed and Atriplex patens achieve the highest mAP@0.5:0.95 values of 90.8% and 89.4%, respectively, indicating that these classes exhibit relatively stable visual patterns and are easier to distinguish from surrounding vegetation. By contrast, Siberia cocklebur, Sonchus wightianus, Setaria viridis, and Convolvulus arvensis show comparatively lower mAP@0.5:0.95 values, suggesting that these categories remain more challenging under stricter localization criteria, despite their high mAP@0.5 scores.

**Table 5:** Class-wise detection performance of CSAG-DETR.

Class name	P/%	R/%	mAP@0.5/%	mAP@0.5:0.95/%
Cirsium arvense	97.2	97.7	99.2	79.4
Convolvulus arvensis	98.3	98.2	99.4	74.1
Atriplex patens	97.5	94.5	98.5	89.4
Thlaspi arvense	98.3	97.0	99.1	75.0
Setaria viridis	96.0	96.9	98.4	73.2
Reed	99.7	98.6	99.5	90.8
Siberia cocklebur	99.5	97.7	99.2	71.2
Chenopodium glaucum	97.2	89.3	97.6	82.1
Sonchus wightianus	99.7	96.9	99.4	72.4
All	98.1	96.3	98.9	78.6

**Figure 13:**
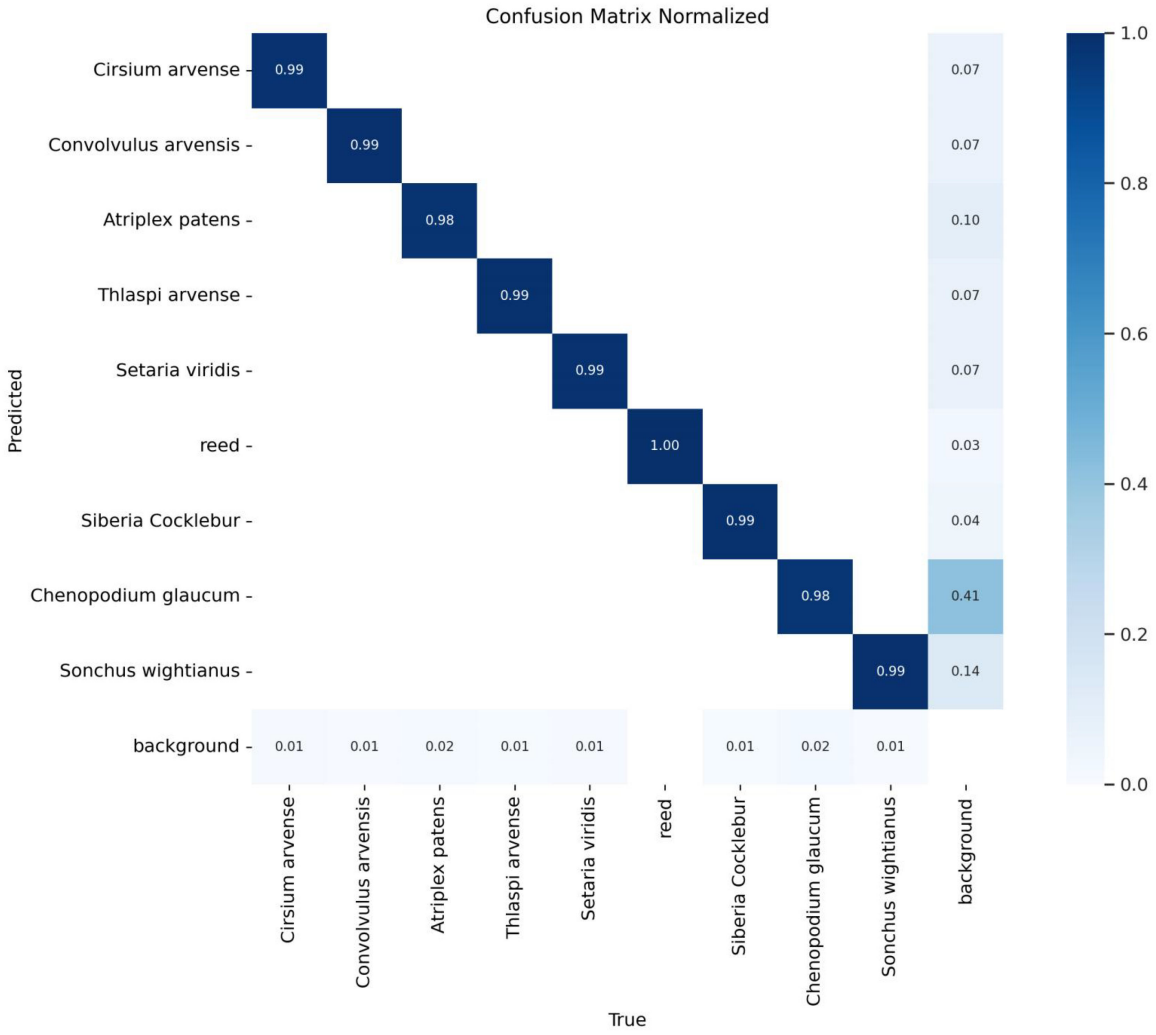
Normalized confusion matrix of the BeetWeed dataset.

Among all categories, Chenopodium glaucum presents the lowest recall of 89.3%, indicating that this class is more prone to missed detections. This phenomenon is consistent with the normalized confusion matrix shown in **Figure 13**, where most weed categories exhibit dominant diagonal responses close to 1.0, demonstrating excellent inter-class separability. However, some background regions are still misclassified as weed targets, particularly for Chenopodium glaucum and, to a lesser extent, Sonchus wightianus and Atriplex patens. This result suggests that visually similar soil textures, plant debris, or shadow patterns may still introduce false positives for certain categories. In addition, the bottom row of the confusion matrix indicates that only a small proportion of true weed instances are predicted as background, confirming that the proposed model maintains a relatively low missed-detection rate across most classes.

Overall, the class-wise results and confusion matrix analysis demonstrate that CSAG-DETR provides highly reliable category discrimination on the BeetWeed dataset. Although several weed categories remain more sensitive to background interference and fine-grained structural ambiguity, the proposed model still achieves strong and stable performance across all categories, highlighting its effectiveness for practical weed detection in complex sugar beet field environments.

### 4.4 Comparison with mainstream object detection algorithms

To rigorously assess the capabilities of the proposed CSAG-DETR, we benchmarked it against a wide array of leading object detectors. The baseline models spanned two-stage frameworks like Faster R-CNN, anchorfree Transformer networks including the RT-DETR series (Zhao et al., 2024) and DEIM (Huang et al., 2025), as well as anchor-based single-stage architectures (YOLOX (Ge et al., 2021), YOLOv5m (Jocher et al., 2022), YOLOv8m (Varghese and M, 2024), YOLOv10m (Chen et al., 2024), YOLOv11m (Li et al., 2023a), and YOLOv12m (Tian et al., 2025)). According to **Table 6**, our method outperforms all competitors on the augmented BeetWeed dataset. It delivers peak accuracies of 78.6% (mAP@0.5:0.95) and 98.9% (mAP@0.5). Furthermore, when evaluated against the robust RT-DETR-R18 baseline, our framework records absolute improvements of +9.6% and +1.4% in mAP@0.5:0.95 and mAP@0.5, respectively. Notably, it secures these superior metrics while maintaining a lean structural size of 14.3M parameters and a computational demand of just 48.1 GFLOPs, highlighting an optimal synergy between detection power and resource economy.

**Table 6:** Comparison of different models on the BeetWeed dataset with data augmentation.

Model	P/%	R/%	FPS	mAP@0.5/%	mAP@0.5:0.95/%	FLOPs/G	Param/M	Weight/MB
Faster R-CNN	48.4	54.8	20.6	92.5	56.2	216.0	41.5	216.0
DEIM	90.5	84.5	112.5	95.3	72.9	24.9	10.2	39.2
YOLOv5m	95.4	94.5	255.3	97.9	75.9	64.0	25.1	96.1
YOLOv8m	95.6	89.3	319.0	96.2	70.2	67.5	23.2	44.6
YOLOv10m	91.0	86.4	160.5	94.1	65.4	58.9	15.4	31.9
YOLOv11m	95.1	90.2	120.1	96.2	68.9	67.7	20.0	38.6
YOLOv12m	96.1	91.8	120.5	97.6	74.5	67.1	20.1	77.5
YOLOX	86.3	81.5	123.4	95.9	63.8	13.4	8.9	68.5
RT-DETR-R18	94.1	94.0	196.6	97.5	69.0	57.0	19.9	77.0
RT-DETR-R34	95.9	90.4	137.9	96.2	67.6	88.8	31.1	119.9
RT-DETR-R50	96.6	90.3	92.6	96.5	66.2	129.6	42.0	163.9
RT-DETR-R101	93.4	88.5	59.9	94.3	63.5	247.1	74.7	292.9
RT-DETR-L	95.8	92.8	109.7	96.2	66.4	103.5	32.0	125.8
CSAG-DETR (ours)	98.1	96.3	167.3	98.9	78.6	48.1	14.3	55.1

When compared to traditional CNN architectures like YOLOv5m and YOLOv8m, CSAG-DETR demonstrates enhanced robustness in recognizing tiny and partially hidden weeds, even in difficult lighting environments. Moreover, the introduction of GAGDU and GAGDD in the feature pyramid neck improves cross-scale feature interaction, mitigating the sampling artifacts that frequently occur in dense and cluttered vegetation environments.

Relative to Transformer-based RT-DETR variants, CSAG-DETR achieves comparable or higher accuracy while simultaneously reducing computational overhead. It operates more efficiently than deeper models such as RT-DETR-R50 and RT-DETR-R101, demonstrating the advantages of the proposed hybrid attention– convolution architecture. Additionally, the qualitative comparisons in **Figure 8** further confirm that CSAG-DETR consistently outperforms other advanced detectors, showing stronger sensitivity to small objects, occlusion, and strong sunlight, as well as better robustness under complex real-world field conditions.

### 4.5 Ablation study

To systematically evaluate the contribution of each proposed component in the CSAG-DETR framework, ablation experiments were conducted based on the RT-DETR-R18 baseline under identical training settings. As summarized in **Table 7**, the baseline model achieved 97.5% mAP@0.5 and 69.0% mAP@0.5:0.95. On this basis, we further investigated the individual and joint effects of CSMN, CSLM, and GAGDU and GAGDD through a series of controlled experiments.

**Table 7:** Ablation study of different module combinations.

Baseline	CSMN	CSLM	GAGDU and GAGDD	P/%	R/%	mAP@0.5/%	mAP@0.5:0.95/%
RT-DETR-R18				94.1	94.0	97.5	69.0
	✓			95.9	96.1	98.0	70.1
		✓		97.8	95.1	98.1	71.9
			✓	97.2	94.9	98.2	74.8
	✓	✓		97.2	95.5	98.2	76.4
	✓		✓	97.2	93.2	97.7	72.3
		✓	✓	95.9	96.1	98.0	70.7
	✓	✓	✓	98.1	96.3	98.9	78.6

When CSMN is introduced alone, precision increases from 94.1% to 95.9%, recall from 94.0% to 96.1%, mAP@0.5 from 97.5% to 98.0%, and mAP@0.5:0.95 from 69.0% to 70.1%. These improvements indicate that the proposed cross-stage multi-scale design effectively strengthens contextual representation and edge-aware feature extraction, thereby improving the detection of small and partially occluded weeds under complex background interference.

When CSLM is added independently, the model achieves 97.8% precision, 95.1% recall, 98.1% mAP@0.5, and 71.9% mAP@0.5:0.95. Compared with the baseline, the gain is particularly evident in precision and high-IoU performance, suggesting that CSLM enhances fine-grained textures and boundary cues and helps reduce false detections in densely vegetated scenes.

When only GAGDU and GAGDD are incorporated, the model reaches 97.2% precision, 94.9% recall, 98.2% mAP@0.5, and 74.8% mAP@0.5:0.95. Among the single-module settings, this configuration yields the largest improvement in mAP@0.5:0.95, demonstrating that globally guided dual-path resampling is particularly effective in improving semantic consistency and structural preservation during cross-scale feature transformation.

The combination of CSMN and CSLM further improves the performance to 97.2% precision, 95.5% recall, 98.2% mAP@0.5, and 76.4% mAP@0.5:0.95. This result confirms the strong complementarity between contextual modeling and local-detail enhancement, indicating that jointly strengthening multi-scale semantics and fine structural representation is beneficial for weed detection in dense field environments.

To further analyze the interaction among modules, two additional dual-module ablations were conducted. When CSMN is combined with GAGDU and GAGDD, the model achieves 97.2% precision, 93.2% recall, 97.7% mAP@0.5, and 72.3% mAP@0.5:0.95. Although this configuration still outperforms the baseline in overall accuracy, its improvement is more limited than that of the CSMN+CSLM setting, suggesting that feature resampling enhancement alone cannot fully compensate for the lack of explicit local-detail reinforcement. Similarly, when CSLM is combined with GAGDU and GAGDD, the model obtains 95.9% precision, 96.1% recall, 98.0% mAP@0.5, and 70.7% mAP@0.5:0.95. This result indicates that local-detail enhancement and attention-guided resampling are beneficial, but their interaction remains constrained without the support of stronger cross-stage contextual modeling.

The complete CSAG-DETR framework, integrating CSMN, CSLM, and GAGDU and GAGDD simultaneously, achieves the best performance across all evaluation metrics, including 98.1% precision, 96.3% recall, 98.9% mAP@0.5, and 78.6% mAP@0.5:0.95. Compared with the baseline, this corresponds to gains of +4.0%, +2.3%, +1.4%, and +9.6%, respectively. These results demonstrate that the three proposed components are highly complementary: CSMN mainly improves multi-scale contextual representation, CSLM strengthens local-detail perception, and GAGDU and GAGDD stabilize cross-scale feature propagation. Their joint integration therefore provides the most robust and discriminative feature representation for sugar beet weed detection.

As illustrated in **Figure 9**, the qualitative comparisons further support the quantitative results. The baseline model is more prone to missed detections and less reliable localization in visually cluttered regions. After introducing CSMN, the model shows improved structural awareness and better discrimination of partially occluded weeds. The addition of CSLM further strengthens sensitivity to fine textures and boundaries, while GAGDU and GAGDD improve feature consistency across scales and reduce ambiguity in difficult regions. Among all configurations, the full CSAG-DETR achieves the most complete and accurate detection results, confirming the effectiveness of the proposed multi-component design under complex agricultural field conditions.

### 4.6 Comparison of experimental results with different backbone networks

To investigate the influence of backbone architecture on detection performance, several lightweight and efficient backbone networks were integrated into the RT-DETR framework for comparative evaluation. As summarized in **Table 8**, different backbones present distinct trade-offs between detection accuracy and computational complexity. EfficientViT, MobileNetV4, and ConvNeXtV2 achieve relatively low computational costs, but their detection accuracy remains limited under challenging conditions involving complex illumination, occlusion, and cluttered backgrounds. Rmt and FasterNet provide moderate improvements in precision and recall due to their stronger feature extraction capability, although this comes with increased computational overhead. EfficientFormerV2 and StarNet show competitive overall performance, achieving more than 97% mAP@0.5 while maintaining a relatively favorable balance between efficiency and accuracy.

**Table 8:** Comparison of experimental results with different backbone networks.

Model	P/%	R/%	mAP@0.5/%	mAP@0.5:0.95/%	FLOPs/G	Param/M	Weight/MB
RT-DETR + EfficientViT (Liu et al., 2023)	94.5	88.3	95.0	66.0	27.3	10.7	42.6
RT-DETR + MobileNetV4 (Qin et al., 2024)	93.2	85.3	93.2	64.2	39.5	11.3	44.4
RT-DETR + Rmt (Fan et al., 2024)	94.1	86.8	93.9	64.5	61.1	21.4	330.3
RT-DETR + FasterNet (Chen et al., 2023)	95.2	92.5	96.8	69.6	49.5	16.8	65.2
RT-DETR + EfficientFormerV2 (Li et al., 2023b)	96.8	93.1	97.4	72.9	29.5	11.8	95.3
RT-DETR + ConvNeXtV2 (Woo et al., 2023)	92.3	86.7	93.8	63.3	31.9	12.3	48.0
RT-DETR + StarNet (Ma et al., 2024)	96.5	93.0	97.2	72.1	31.8	12.0	46.9
RT-DETR + CSMN(ours)	95.9	96.1	98.0	70.1	58.2	19.9	76.2

In contrast, the proposed CSMN-integrated RT-DETR achieves the best mAP@0.5 of 98.0% and the highest recall of 96.1% among all evaluated backbone settings, indicating its superior ability to capture discriminative multi-scale and structural features in sugar beet weed detection. Although its mAP@0.5:0.95 is slightly lower than that of EfficientFormerV2, the proposed backbone shows stronger overall detection robustness in dense and visually complex agricultural scenes. These results suggest that CSMN effectively enhances cross-stage feature interaction and fine-grained structural representation, thereby improving the detection of small, overlapping, and visually similar weeds. Overall, the findings confirm that backbone architecture plays a critical role in balancing detection accuracy and computational efficiency for real-time agricultural vision tasks.

### 4.7 Generalization evaluation

#### 4.7.1 Generalization evaluation under adverse conditions

**Figure 14:**
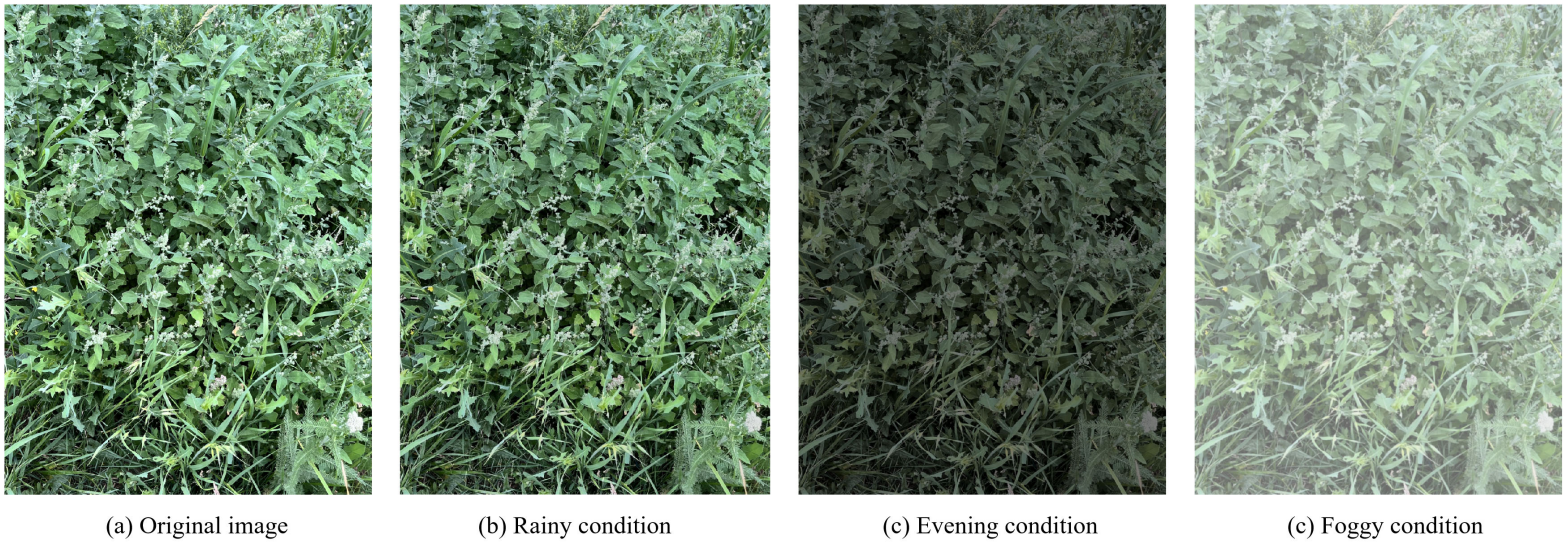
Visual examples of different environmental conditions used for generalization testing. From left to right: **(a)** original image, **(b)** rainy condition, **(c)** evening condition, and **(d)** foggy condition (all simulated via data augmentation).

To comprehensively evaluate the robustness and generalization capability of the proposed model in complex agricultural environments, additional experiments were conducted under simulated adverse weather and lighting conditions. As illustrated in **Figure 14**, three types of environmental degradations were introduced into the BeetWeed dataset to emulate real-world field challenges: rainy, foggy, and evening (low-light) scenarios.

The rainy condition was simulated by generating synthetic streak noise with varying densities and slant angles to mimic natural rainfall. Foggy conditions were produced using a depth-aware Gaussian haze model that reduces scene contrast and obscures visual boundaries. The evening condition was simulated by decreasing image brightness and saturation to replicate low-illumination environments. These augmentations introduce realistic variations in visibility and appearance, enabling a rigorous assessment of model performance under unfavorable field conditions.

The results in **Table 9** show that adverse weather and lighting degrade performance across all models to varying degrees. Rainy conditions primarily blur edges and introduce streak noise, leading to notable drops in recall among most RT-DETR variants. Fog induces the most severe performance decline, as reduced contrast and boundary ambiguity significantly lower mAP@0.5:0.95. Low-light (evening) conditions also negatively affect accuracy due to reduced visibility and color distortion.

**Table 9:** Detection results under different weather and lighting conditions.

Model	P/%	R/%	mAP@0.5	mAP@0.5:0.95
Experimental results in rainy conditions				
RT-DETR-R18	96.7	92.3	97.1	69.2
RT-DETR-R34	95.5	91.2	96.5	67.8
RT-DETR-R50	95.1	92.5	96.3	66.8
RT-DETR-R101	93.6	88.8	94.6	63.1
RT-DETR-L	96.6	93.4	97.1	70.6
CSAG-DETR (ours)	97.4	93.9	98.0	73.1
Experimental results in evening conditions				
RT-DETR-R18	95.1	91.0	96.1	68.1
RT-DETR-R34	95.6	91.7	96.5	67.6
RT-DETR-R0	95.6	91.8	96.2	67.2
RT-DETR-R101	93.6	87.4	94.1	62.6
RT-DETR-L	96.2	88.1	95.2	65.1
CSAG-DETR (ours)	96.9	92.8	97.5	72.5
Experimental results in foggy conditions				
RT-DETR-R18	94.9	90.9	96.2	69.1
RT-DETR-R34	95.8	90.2	96.0	68.1
RT-DETR-R50	95.0	90.2	95.6	66.0
RT-DETR-R101	93.3	87.7	90.2	63.1
RT-DETR-L	96.9	93.3	97.2	71.1
CSAG-DETR (ours)	97.0	94.7	97.9	72.7

Despite these challenges, CSAG-DETR consistently achieves the highest performance under all three adverse conditions. Under rainy, foggy, and evening scenarios, it obtains the highest mAP@0.5 and mAP@0.5:0.95. This superior robustness is attributed to the synergistic effects of the CSMN, CSLM, GAGDU and GAGDD modules, which together improve multi-scale continuity, fine-detail preservation, and globally attentive feature alignment.

Overall, the results demonstrate that the proposed model maintains stable detection accuracy and strong generalization capability even under severe visual degradations, highlighting its potential for reliable real-world deployment across diverse and challenging agricultural environments.

#### 4.7.2 Generalization evaluation on other weed detection datasets

To further examine whether the proposed architectural design remains effective beyond the BeetWeed domain, we conducted an additional cross-dataset evaluation on the publicly available CottonWeedDet12 dataset. Specifically, CottonWeedDet12 was collected at the Mississippi State University Research Farm from June to September 2021 and contains 5,648 images with resolutions ranging from 357 × 353 to 6000×4000 pixels. It includes 9,373 annotated bounding boxes covering 12 plant categories in cotton-field scenes, namely cotton and 11 distinct weed species: waterhemp, morning glory, purslane, spotted spurge, barnyard grass, ragweed, carpetweed, prickly sida, palm amaranth, goose grass, and horsenettle.

Compared with our sugar beet weed detection scenario, CottonWeedDet12 introduces several pronounced domain shifts. First, the crop context changes from sugar beet to cotton, altering the canopy structures, row patterns, leaf morphology, and crop–weed spatial relationships. Second, the weed taxonomy differs substantially between the two datasets, which places a higher premium on transferable fine-grained texture and boundary representations rather than the mere memorization of dataset-specific appearances. Third, the geographic and environmental conditions differ markedly, with CottonWeedDet12 originating from Mississippi, USA, whereas BeetWeed was collected in Xinjiang, China; this divergence introduces significant variations in soil color, illumination, humidity, background vegetation, and field management practices. Finally, the image resolution range and object scale distribution in CottonWeedDet12 are highly heterogeneous, further testing whether the detector can maintain stable localization under severe scale variations and cluttered field backgrounds.

As shown in **Table 10**, CSAG-DETR maintains the best overall accuracy–efficiency trade-off on CottonWeedDet12. It achieves the highest precision, mAP@0.5, and mAP@0.5:0.95, reaching 95.4%, 97.1%, and 94.7%, respectively. Compared with the strongest baseline, RT-DETR-R101, CSAG-DETR improves mAP@0.5 by 0.5 percentage points and mAP@0.5:0.95 by 1.9 percentage points. Meanwhile, compared with the lightweight RT-DETR-R18, it reduces FLOPs from 57.0 G to 48.1 G, parameters from 19.9 M to 14.3 M, and model weight from 77.0 MB to 55.1 MB, corresponding to substantial reductions of 15.6%, 28.1%, and 28.4%, respectively. These results indicate that the architectural merits of CSAG-DETR successfully translate into enhanced performance and deployment efficiency across drastically different agricultural scenarios.

**Table 10:** Comparison results on the CottonWeedDet12 dataset.

Model	P/%	R/%	mAP@0.5/%	mAP@0.5:0.95/%	FLOPs/G	Param/M	Weight/MB
RT-DETR-R18	94.2	92.2	95.8	91.8	57.0	19.9	77.0
RT-DETR-R34	93.6	93.1	96.2	92.6	88.8	31.1	119.9
RT-DETR-R50	94.1	92.1	96.2	92.5	129.6	42.0	163.9
RT-DETR-R101	93.7	93.1	96.6	92.8	247.1	74.7	292.9
RT-DETR-L	93.9	90.8	95.2	91.2	103.5	32.0	125.9
CSAG-DETR	95.4	92.5	97.1	94.7	48.1	14.3	55.1

It is also worth noting that CSAG-DETR does not yield the peak recall on CottonWeedDet12, falling 0.6 percentage points short of the best-performing RT-DETR-R34 and RT-DETR-R101. This implies that unfamiliar weed morphologies and complex cotton-field backgrounds can still induce missed detections, particularly under high inter-class similarity or severe partial occlusion. Nevertheless, the consistently higher precision and mAP scores confirm that CSAG-DETR provides more reliable localization and classification quality overall.

### 4.8 Analysis of experimental results

First, the empirical data presented in **Table 4** and **Figure 7** substantiates the value of the adopted data augmentation methodology for identifying sugar beet weeds. In the absence of these augmentations, the standard RT-DETR-R18 struggles to generalize across fluctuating lighting, varied backgrounds, and target occlusions. By integrating cutout-based occlusion modeling with photometric and geometric transformations, the framework registers significant boosts in mAP@0.5, mAP@0.5:0.95, precision, and recall. These enhancements prove that our augmentation pipeline successfully diversifies the training data and fortifies the detector’s resilience in harsh agricultural settings, all without imposing any extra inference burden.

Second, an analysis of the class-specific metrics in **Table 5** alongside the normalized confusion matrix in **Figure 13** reveals deeper insights into the model’s behavior across different weed types. While CSAG-DETR maintains robust and consistent accuracy overall, performance variations between categories are evident. Weeds with distinct morphological traits, such as reed and Atriplex patens, secure higher mAP@0.5:0.95 scores. Conversely, species like Convolvulus arvensis, Setaria viridis, Sonchus wightianus, and Siberia cocklebur prove more difficult to detect under rigorous localization thresholds. Notably, Chenopodium glaucum yields the lowest recall rate—a finding mirrored in the confusion matrix—highlighting its vulnerability to missed detections or misclassification against visually similar backgrounds. This indicates that environmental clutter and fine-grained structural similarities are still primary hurdles in real-world field detection.

Third, the superiority of the CSAG-DETR architecture on the BeetWeed dataset is explicitly confirmed by the benchmark evaluations in **Table 6** and visual assessments in **Figure 8**. In numerical terms, our proposed network secures the top mAP@0.5 and mAP@0.5:0.95 scores among all evaluated methods, perfectly bridging the gap between inference pace, computational load, and accuracy. When contrasted with the RT-DETR-R18 baseline, CSAG-DETR elevates the mAP@0.5 from 97.5% to 98.9% and the mAP@0.5:0.95 from 69.0% to 78.6%, simultaneously trimming down the parameter count and FLOPs. From a visual perspective, it exhibits exceptional robustness when processing intricate lighting, heavy occlusion, dense foliage, and miniature targets, generating fewer false negatives and positives than competing Transformer-based and YOLO models.

Fourth, the ablation experiments in **Table 7** and corresponding qualitative visualizations in **Figure 9** systematically decode the individual contributions of the integrated modules. Specifically, CSMN advances edge-aware feature interactions and multi-scale contextual mapping; CSLM secures boundary details and enriches local representations; and the GAGDD/GAGDU modules ensure structural integrity and semantic alignment during cross-scale transformations. While each component independently boosts performance, their combined application delivers optimal outcomes. The synergy between CSLM and CSMN is particularly highly complementary, pushing the complete CSAG-DETR model to achieve peak mAP, precision, and recall metrics. This confirms that rather than creating redundancy, these modules collaboratively refine the network via feature resampling, local-detail amplification, and contextual modeling.

Furthermore, findings in **Table 8** underscore the profound impact that backbone selection has on the RT-DETR architecture. Yielding the highest recall and mAP@0.5 of all tested configurations, the customized RT-DETR + CSMN backbone demonstrates a superior capacity for extracting structural and discriminative multi-scale features. Even though the EfficientFormerV2 setup slightly edges out in mAP@0.5:0.95, the CSMN-driven backbone exhibits greater overall stability in visually chaotic and dense field scenarios. These results reiterate the crucial role of backbone engineering in harmonizing computational economy with feature representation for real-time farming applications.

Finally, evaluations across **Figure 14**; **Tables 9**, **Figure 7** validate the model’s remarkable adaptability. When subjected to artificial low-visibility scenarios like dusk, fog, and rain, CSAG-DETR reliably beats all other RT-DETR variants, preserving high detection standards despite weather-related visual degradation. Furthermore, on the CottonWeedDet12 dataset, our framework captures the highest mAP scores while consuming less computational power and fewer parameters than baseline RT-DETR models. Collectively, these outcomes prove our model’s profound resilience to shifting domains, diverse weed species, and suboptimal imaging conditions, underlining its immense value for practical deployment in dynamic agricultural environments.

### 4.9 Comparison with existing crop weed identification approaches

The superiority of our CSAG-DETR in challenging agricultural settings becomes particularly evident when benchmarked against other recent DETR-driven detection systems. To illustrate, the SCR-DETR architecture developed by Zhang et al. (2025b) successfully processed the cotton–weed dataset using a combination of the CGSR framework, a USAR module, and an SRC-integrated backbone. Similarly, Yan et al. (2024) designed the DETR-RPC-CDF, a streamlined weed identifier featuring a collection-distribution feature fusion (CDF) strategy alongside re-parameterized partial convolution (RPC) within its backbone. When evaluated on the Fine24 dataset, their approach outperformed standard techniques by yielding a 2% increase in mAP@0.5, alongside massive efficiency gains: a 43% drop in FLOPs, a 40% reduction in parameter count, and a 40% boost in inference speed.

However, these models primarily focus on relatively simple agricultural scenarios, which differ significantly from the complex field environments considered in this study. Additionally, most of these models and datasets are not publicly accessible, limiting objective comparison and reproducibility. In contrast, the proposed CSAG-DETR is tailored for real-world agricultural conditions characterized by dense occlusions, background clutter, and large-scale variations. By effectively addressing these challenges, CSAG-DETR demonstrates clear novelty and practical value for deployment in complex field applications.

### 4.10 Limitations of this study

Limitations and Challenges. It is important to acknowledge certain constraints within this research. The efficacy of deep learning frameworks is intrinsically linked to the volume and integrity of training datasets. While our 3,802 source images were expanded to 18,990 via varied augmentation techniques—boosting generalization—these synthetic samples cannot entirely replicate the intricate interplay of background, occlusion, and lighting found in natural fields. Furthermore, specific augmentations, such as adjusting saturation or brightness, may introduce distributional shifts that don’t perfectly align with real-world scenarios, potentially causing performance variance during field use.

Geographically, our data was sourced solely from Xinjiang, China, which may limit the model’s adaptability to sugar beet crops in different ecological zones. The current dataset also focuses on later growth phases, potentially reducing accuracy during the seedling or middle growth periods. Moreover, although we simulated adverse weather (e.g., fog, rain, and night) to improve resilience, these artificial models simplify complex physical phenomena like humidity-driven reflectance and light scattering.

Future Directions. Future research will pursue three primary avenues: (1) expanding the geographic and seasonal scope of data collection to bolster cross-regional adaptability; (2) incorporating a wider variety of weed species and early-stage crop samples to resolve growth-stage limitations; and (3) migrating the optimized model onto mobile or embedded hardware for rigorous on-site validation in actual farming environments.

## 5 Conclusion

Weeds impose a substantial negative effect on both the yield and sugar accumulation of sugar beet, and accurate weed detection remains a key prerequisite for the practical deployment of precision weeding systems. To address this challenge, this study advances intelligent sugar beet weed detection from both dataset construction and model design perspectives. Specifically, we constructed a dedicated BeetWeed dataset with TXT-format annotations, consisting of 3,802 RGB images covering nine common weed species. To improve model robustness under complex field conditions, including illumination variation, plant occlusion, background clutter, and scale inconsistency, multiple data augmentation strategies were introduced. In addition, the dataset was compressed by more than 90%, which substantially reduced storage requirements, training cost, and processing time without noticeably affecting detection performance.

Experimental results demonstrate that the proposed augmentation strategy significantly improves the generalization ability of the baseline detector. After augmentation, the model achieves 97.5% mAP@0.5 and 69.0% mAP@0.5:0.95, while precision and recall increase from 81.2% and 71.3% to 94.1% and 94.0%, respectively. These results confirm that task-oriented augmentation plays an important role in improving detection robustness for real-world sugar beet weed identification.

Building upon this foundation, we further propose CSAG-DETR, a lightweight real-time weed detection framework based on RT-DETR-R18. By introducing the Cross-Stage Multi-Scale Network (CSMN), the model enhances multi-scale contextual representation and sensitivity to fine-grained structural details. The Cross-Stage Local-Detail Module (CSLM) further strengthens local texture modeling and boundary preservation, while the Global Attention-Gated Dual-Path Upsampler (GAGDU) and Downsampler (GAGDD) improve semantic consistency and structural fidelity during cross-scale feature transitions. Through the joint effect of these modules, CSAG-DETR achieves more robust and discriminative feature representation for weed detection in complex sugar beet field environments.

Extensive experiments demonstrate the effectiveness of the proposed framework. On the BeetWeed dataset, CSAG-DETR achieves 98.9% mAP@0.5 and 78.6% mAP@0.5:0.95, outperforming 13 mainstream detection models while maintaining favorable efficiency. Additional evaluations under adverse conditions and on the public CottonWeedDet12 dataset further confirm its strong robustness and generalization capability. Overall, the proposed dataset and lightweight detection framework jointly provide a useful foundation for intelligent and field-adaptable weed detection in precision agriculture.

Despite these encouraging results, several limitations still remain. The current dataset is region-specific and does not yet fully cover multi-regional, multi-seasonal, or early-growth-stage conditions. In future work, we will further expand the dataset, improve the balance between detection accuracy and real-time efficiency, and validate the proposed method through in-field experiments on embedded or mobile agricultural platforms.

## Data Availability

The raw data supporting the conclusions of this article will be made available by the authors, without undue reservation.
